# Altered brain metabolites in male nonhuman primate offspring exposed to maternal immune activation

**DOI:** 10.1016/j.bbi.2024.07.011

**Published:** 2024-07-18

**Authors:** Richard J. Maddock, Roza M. Vlasova, Shuai Chen, Ana-Maria Iosif, Jeffrey Bennett, Costin Tanase, Amy M. Ryan, Takeshi Murai, Casey E. Hogrefe, Cynthia D. Schumann, Daniel H. Geschwind, Judy Van de Water, David G. Amaral, Tyler A. Lesh, Martin A. Styner, A. Kimberley McAllister, Cameron S. Carter, Melissa D. Bauman

**Affiliations:** a Department of Psychiatry and Behavioral Sciences, School of Medicine, University of California Davis, Sacramento, CA, USA; b Department of Psychiatry, University of North Carolina, Chapel Hill, NC, USA; c Division of Biostatistics, Department of Public Health Sciences, School of Medicine, University of California Davis, Sacramento, CA, USA; d California National Primate Research Center, University of California Davis, Davis, CA, USA; e Neurogenetics Program, Department of Neurology, University of California Los Angeles, Los Angeles, CA, USA; f Rheumatology/Allergy and Clinical Immunology, School of Medicine, University of California Davis, Sacramento, CA, USA; g MIND Institute, School of Medicine, University of California Davis, Sacramento, CA, USA; h Department of Computer Science, University of North Carolina, Chapel Hill, NC, USA; i Center for Neuroscience, University of California Davis, Davis, CA, USA; j Physiology and Membrane Biology, School of Medicine, University of California Davis, Sacramento, CA, USA

**Keywords:** Gestational immune activation, Resilience, Macaque, Magnetic resonance spectroscopy, Childhood, Adolescence, N-acetylaspartate, Myo-inositol, Prefrontal

## Abstract

Converging data show that exposure to maternal immune activation (MIA) in utero alters brain development in animals and increases the risk of neurodevelopmental disorders in humans. A recently developed non-human primate MIA model affords opportunities for studies with uniquely strong translational relevance to human neurodevelopment. The current longitudinal study used 1H-MRS to investigate the developmental trajectory of prefrontal cortex metabolites in male rhesus monkey offspring of dams *(n* = 14) exposed to a modified form of the inflammatory viral mimic, polyinosinic:polycytidylic acid (Poly IC), in the late first trimester. Brain metabolites in these animals were compared to offspring of dams that received saline (*n* = 10) or no injection (*n* = 4). N-acetylaspartate (NAA), glutamate, creatine, choline, myo-inositol, taurine, and glutathione were estimated from PRESS and MEGA-PRESS acquisitions obtained at 6, 12, 24, 36, and 45 months of age. Prior investigations of this cohort reported reduced frontal cortical gray and white matter and subtle cognitive impairments in MIA offspring. We hypothesized that the MIA-induced neurodevelopmental changes would extend to abnormal brain metabolite levels, which would be associated with the observed cognitive impairments. Prefrontal NAA was significantly higher in the MIA offspring across all ages (*p* < 0.001) and was associated with better performance on the two cognitive measures most sensitive to impairment in the MIA animals (both *p* < 0.05). Myo-inositol was significantly lower across all ages in MIA offspring but was not associated with cognitive performance. Taurine was elevated in MIA offspring at 36 and 45 months. Glutathione did not differ between groups. MIA exposure in male non-human primates is associated with altered prefrontal cortex metabolites during childhood and adolescence. A positive association between elevated NAA and cognitive performance suggests the hypothesis that elevated NAA throughout these developmental stages reflects a protective or resilience-related process in MIA-exposed offspring. The potential relevance of these findings to human neurodevelopmental disorders is discussed.

## 1. Introduction

Converging evidence from epidemiological and preclinical studies has demonstrated that a variety of viral or bacterial infections leading to activation of the maternal immune response during pregnancy constitute a significant risk for altered brain development and neurodevelopmental disorders (NDD) in the offspring (Smith et al., 2007; Estes and McAllister, 2016; Brown and Meyer, 2018; Han et al., 2021). The evidence that immune signaling molecules play a crucial role in orchestrating all stages of brain development highlights a mechanism by which maternal immune activation (MIA) could interfere with normal neurodevelopment in utero (Estes and McAllister, 2014; 2015; 2016; Gumusoglu and Stevens, 2019).

Animal models of MIA can be a valuable tool for investigating the effects of gestational immune activation on neurodevelopment (Estes and McAllister, 2016; Bauman and Van de Water, 2020; Hanson et al. 2022). A widely used model activates the immune system with polyinosinic:polycytidylic acid (Poly IC). This synthetic double-stranded RNA molecule mimics the double-stranded RNA found in many viral pathogens (Alexopoulou et al. 2001). The Poly IC MIA model has been used to study brain development in several different species, including mice and rats (Estes and McAllister, 2016; Kentner et al., 2019), and more recently ferrets (Li et al., 2018), pigs (Rymut et al., 2020), and nonhuman primates (NHPs) (Bauman et al., 2014; Ryan and Bauman, 2022). The translational potential for understanding human NDDs is particularly promising with Poly IC MIA models in NHPs (Ryan and Bauman, 2022). Recent studies from our group using this model system have demonstrated abnormalities relevant to schizophrenia and other NDDs, including impairments in social behavior and cognition, elevated striatal dopamine, abnormal dendritic structure and reduced gray matter density in prefrontal regions, and elevated extracellular free water in the cingulate cortex (Bauman et al., 2014; Vlasova et al., 2021; Bauman et al., 2019; Smucny et al., 2023; Hanson et al., 2023; Lesh et al., 2023).

Proton magnetic resonance spectroscopy (1H-MRS) is a non-invasive method for estimating the level of specific metabolites in the brain that may provide additional insight into mechanisms of pathogenesis and resilience following exposure to MIA (Rae, 2014). The most commonly measured neurometabolites include n-acetylaspartate (NAA), glutamate, creatine + phosphocreatine (“creatine”), choline-containing compounds glycerophosphocholine and phosphocholine (“choline”), and myo-inositol. NAA is synthesized almost exclusively in neuronal mitochondria and often reflects the integrity of neuronal metabolism (Moffett et al., 2007; Maddock and Buonocore, 2012). One important function of NAA is to supply acetate moieties to oligodendrocytes in support of myelin synthesis (Chakraborty et al., 2001). Glutamate is a ubiquitous intracellular metabolite and the most widespread neurotransmitter in the mammalian brain (Meldrum, 2000). 1H-MRS glutamate measures may reflect glutamatergic neurotransmission capacity (Stanley and Raz, 2018). Creatine and phosphocreatine serve to distribute metabolic energy in the form of phosphate bonds within all brain cells (Maddock and Buonocore, 2012). Glycerophosphocholine and phosphocholine have important roles in brain phospholipid metabolism and maintenance of cell membranes (Klein, 2000; Maddock and Buonocore, 2012; De Freitas Saito et al., 2022). Myo-inositol, as a non-perturbing osmolyte, has a major role in cell volume regulation (Fisher et al., 2002). Although not specific to neuroinflammation, elevations in myo-inositol and choline are observed in some conditions characterized by neuroinflammation, including neuroviral infections (Oriolo et al., 2018; Chelala et al., 2020) and experimental models of glial activation (Kim et al., 2005; Ligneul et al., 2019). Other important neurometabolites, including GABA, glutathione, and taurine, can be measured using specialized acquisition methods or, in the case of taurine, in species with higher brain taurine levels than in humans. 1H-MRS measures of GABA may reflect the capacity for GABAergic neurotransmission (Yoon et al., 2010). Glutathione is a major antioxidant in the brain and reduced levels are seen in several neuroinflammatory conditions (e.g., HIV, multiple sclerosis) (Buhl et al., 1989; Carvalho et al., 2014) and in association with elevated gray matter free water in schizophrenia (Lesh et al., 2019). Taurine, which exhibits higher brain levels in rodents and monkeys than in humans (Sturman, 1986), has antioxidant, osmoregulating, neuromodulating, and immune-modulating effects in the brain (Oja and Saransaari, 2017).

Only two prior studies have used 1H-MRS to examine the effects of MIA on brain metabolites in animal models (mouse and rat). Li et al. (2015) found significantly elevated n-acetylaspartate (NAA) and significantly reduced myo-inositol in the anterior cingulate cortex of adult offspring of MIA mice. Vernon et al. (2015) found significantly reduced GSH and taurine in the prefrontal cortex of adult but not adolescent offspring of MIA rats. They also observed a significant age-by-MIA interaction for prefrontal NAA levels, suggesting a greater drop in NAA from adolescence to adulthood in MIA rats. The current study is the first to examine brain metabolites using 1H-MRS in NHP offspring exposed to Poly IC MIA. Using both conventional and spectral editing methods, we aimed to examine the impact of MIA on brain metabolites potentially reflecting antioxidant capacity and inflammation (GSH, myo-inositol, choline, and taurine), neuronal integrity (NAA), osmotic balance (myo-inositol, choline, and taurine) and capacity for glutamatergic neurotransmission (glutamate). To better understand the pathophysiological significance of any neurometabolic abnormalities identified in MIA offspring, their associations with measures sensitive to MIA-induced cognitive impairment documented in the same animals (Vlasova et al., 2021) were also examined.

## 2. Materials and methods

### 2.1. Overview

Experimental procedures were developed in collaboration with the veterinary, animal husbandry, and environmental enrichment staff at the California National Primate Research Center (CNPRC) and approved by the University of California, Davis Institutional Animal Care and Use Committee. All attempts were made to promote the psychological and social well-being of animals participating in this research. This included social housing, an enriched diet, positive reinforcement strategies, and minimizing the duration of daily training/testing sessions (Vlasova et al., 2021).

### 2.2. Animal selection and MIA procedures

Animals in this study represent the same cohort previously reported on by our group (Vlasova et al., 2021; Lesh et al., 2023; Smucny et al., 2023). Consequently, some methods are presented in condensed form. Pregnant dams aged 5 to 12 years were selected (based on age, weight, parity, and number of live births) from the indoor time-mated breeding colony at the CNPRC and assigned to MIA (*n* = 14) and control (*n* = 10 saline-treated and *n* = 4 untreated) groups. Synthetic double-stranded RNA (polyinosinic:polycytidylic acid [Poly IC] stabilized with poly-L-lysine [Poly ICLC]) (Oncovir, Inc.; 0.25 mg/kg i.v.) or sterile saline (equivalent volume to Poly ICLC) was injected at 07:00 h in the cephalic vein in awake animals in the late first trimester (gestational days (GD) 43, 44, and 46). Blood samples collected 6 h after the second (GD 44) and third (GD 46) Poly ICLC injections confirmed a strong proinflammatory cytokine response as evidenced by the change in serum interleukin-6 (IL-6) from baseline samples as previously described (Vlasova et al., 2021). Following recent guideline recommendations for improved reporting of MIA model methods, the Kentner et al. (2019) checklist is provided in the Supplement. One offspring from the MIA group was euthanized at 6 months and a second at 42 months of age, in both cases due to unrelated health conditions. Thus, these animals contributed data only at the 6-month time point and the 6, 12, 24, and 36-month time points, respectively.

### 2.3. Rearing conditions and husbandry

Infants were raised in individual cages with their mothers, where they always had visual access to other mother-infant pairs. For three hours each day, each mother and infant pair had free access to a familiar social group, including an adult male and three other mother-infant pairs. As previously described, additional socialization, rearing, and husbandry procedures were also applied (Vlasova et al., 2021).

### 2.4. Neuroimaging

MRI and MRS were acquired at 6, 12, 24, 36, and 45 months of age using a Siemens Skyra 3-T system with an 8-channel coil optimized for monkeys (Vlasova et al., 2021). Animals were sedated with ketamine for tracheal intubation and then anesthetized with isoflurane, which was maintained at 1.3 % to 2.0 % throughout scanning. Two animals were found to be sensitive to isoflurane and were anesthetized with propofol instead. One animal initially showed isoflurane sensitivity and received propofol for the six-month scan but was tested and cleared to receive isoflurane for the remaining time points. The propofol infusion rate was varied to maintain the animal at a steady state of anesthesia. All animals were positioned in an MR-compatible stereotaxic apparatus, to which the 8-channel receiver coil was attached. The coil was positioned and the scanner table landmarked so the center of the animal’s brain was at the isocenter of the MRI field.

**Fig. 1.**
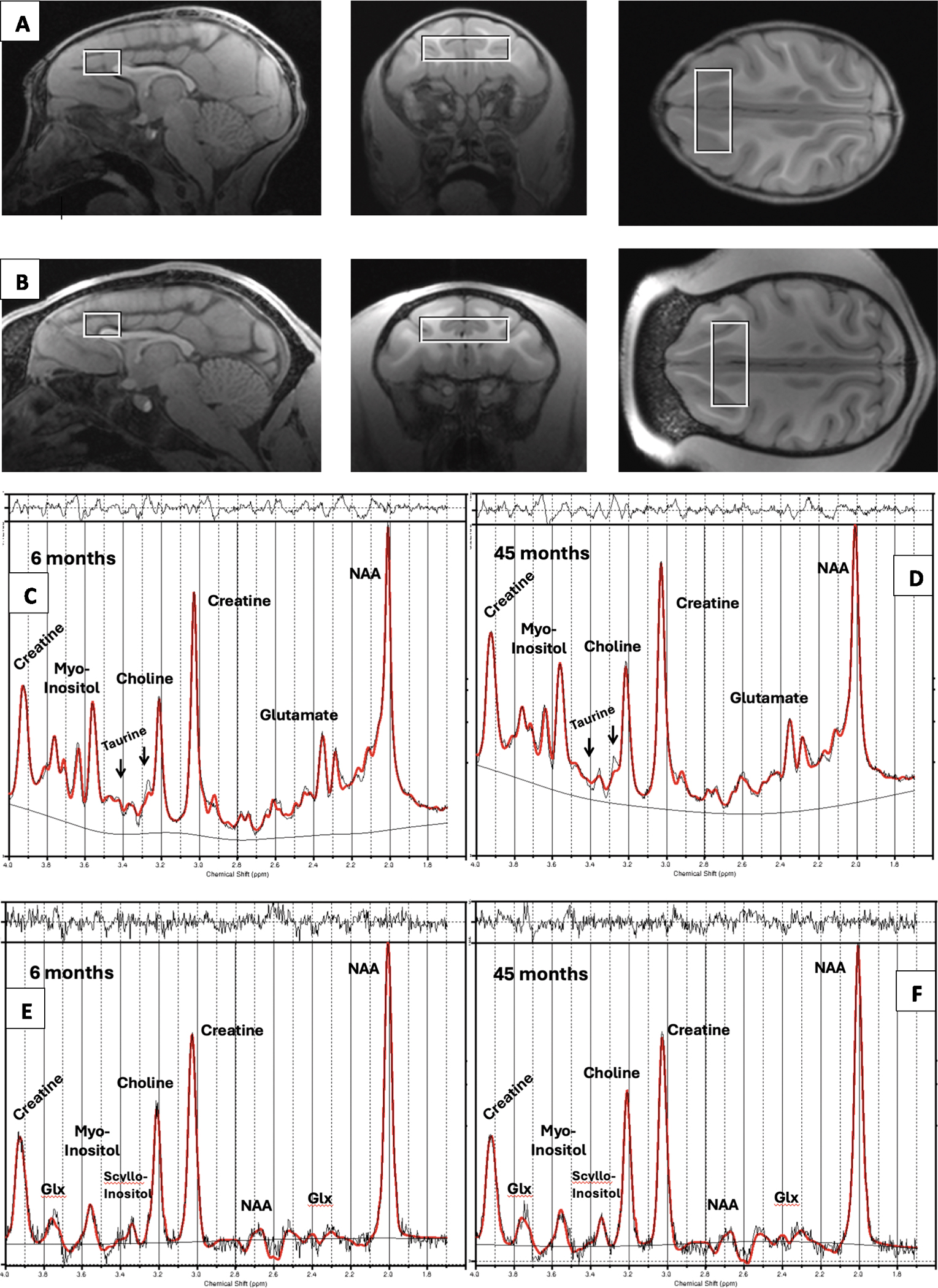
An example of voxel locations from the same animal (in MIA group) at age 6 months (row A) and 45 months (row B). The spatial scaling of the brain and voxel is equal at both ages. An example PRESS spectrum (TE/TR 33/1500) from the same animal is shown at 6 months (panel C) and 45 months of age (panel D). An example off-resonance MEGA-PRESS spectrum (TE/TR 131/2000) from the same animal is shown at 6 months (panel E) and 45 months of age (panel F). Smoother, thick red lines are LCModel fits. Lighter black lines are raw data. Wavy lines under spectrum are estimated baselines. Residual signal is shown above each spectrum.

T1-weighted images (TR=2500 ms, TE=3.65 ms, flip angle = 7°, FOV 256 × 256) were acquired as 480 sagittal slices with voxel dimensions of 0.6 × 0.6 × 0.6 mm. Acquired images were interpolated during image reconstruction to 512 × 512 voxels with a final resolution of 0.3 × 0.3 × 0.3 mm. T2-weighted images (TR=3000 ms, TE=308 ms, FOV 256 × 256 were acquired as 240 sagittal slices with voxel dimensions of 0.8 × 0.8 × 0.8 mm, which were interpolated during reconstruction to 512 × 512 voxels with a final resolution of 0.4 × 0.4 × 0.4 mm. The T1-weighted images were used to guide placement of a 2.5 × 0.7 × 1.0 cm voxel in the bilateral prefrontal cortex from which MRS data were collected. The voxel was centered on the midline at the level of the genu of the corpus callosum, with its posterior border crossing the anterior tip of the genu (Fig. 1).

All metabolite data except glutathione (GSH) were acquired with a water-suppressed PRESS sequence (TR=1500 ms, TE=33 ms, NEX=160, bandwidth = 1000 Hz, eight-step phase cycling, delta frequency −1.7, total duration = 4.1 min). GSH data were acquired using a water-suppressed MEGA-PRESS sequence adapted for GSH (Mescher et al., 1998; An et al., 2009; Nezhad et al., 2017; Lesh et al., 2019). Parameters included TR=2000 ms, TE=131 ms, bandwidth = 2000 Hz, eight-step phase cycling, delta frequency −1.7, edit pulse on = 4.56 ppm, edit pulse off = 4.9 ppm, edit pulse bandwidth = 30 Hz. The edited spectra were acquired in two sequential acquisitions of 176 averages (total 352 averages, 11.7 min total duration). Non-water-suppressed acquisitions were acquired immediately after the PRESS scan and between the two MEGA-PRESS acquisitions.

All image and metabolite data were processed by operators unaware of group assignment. Metabolite data from PRESS spectra were quantified using LCModel, version 6.3–1L (Provencher, 2001) with a basis set matched to the scanning parameters provided with LCModel (consisting of: creatine, phosphocreatine, n-acetylaspartate (NAA), n-acetylaspartylglutamate, phosphocholine, glycerophosphorylcholine, myoinositol, glutamate, glutamine, glutathione, scyllo-inositol, aspartate, taurine, GABA, and glucose), and an analysis window from 4.0 to 1.7 ppm. GSH data from the MEGA-PRESS spectra were quantified using LCModel, jMRUI 5.2 (Stefan et al., 2009), and custom software in an operator-independent sequence of processing steps, as previously described (Lesh et al., 2019). Briefly, on– and off-resonance spectra from each of the two acquisitions were phase-aligned with LCModel and then zero-filled (2x) and apodized in jMRUI. The on and off spectra were frequency-aligned using custom software and subtracted to generate difference spectra. The difference spectra from the two acquisitions were summed, and the edited GSH cysteine resonance at 2.95 ppm was quantified by linewidth-optimized peak integration.

Anatomical images were used to segment the spectroscopy voxel as follows. First, T1-weighted images were aligned into common space (Shi et al., 2016), bias field corrected, and brain-masked using AutoSeg_3.3.2 (Wang et al., 2014). The masks were manually corrected if necessary. Following this preprocessing, T1-weighted images were segmented into GM, WM, and cerebrospinal fluid (CSF) using NeosegPipeline_v1.0.8 (Cherel et al., 2015). The segmentation results were visually quality-controlled and backpropagated to the native subjects’ space to segment the spectroscopy voxel. The unsuppressed water signal and segmentation values for each animal at each age were used to calculate partial volume corrected water-normalized metabolite values for NAA, total creatine (tCr), total choline (tCho), glutamate, and myo-inositol from the PRESS spectra and GSH from the MEGA-PRESS spectra. Water normalization incorporated corrections for the fractional volumes of CSF, gray matter, and white matter in the voxel as described in the equation below based on the consensus paper by Near et al. (2021).

$$M=S_{M}/S_{W}*1/\left(1-f_{csf}\right)*\left(f_{gm}d_{gm}+f_{wm}d_{wm}+f_{csf}d_{csf}\right)*2/N_{M}*[W]$$

where M= metabolite value, $S_{M}=$ metabolite signal, $S_{W}=$ water signal, $f_{csf}=$ fractional volume of CSF in the voxel, $f_{gm}=$ fractional volume of gray matter in the voxel, $f_{wm}=$ fractional volume of white matter in the voxel, $d_{gm}=$ water content of gray matter (here 43.3), $d_{wm}=$ water content of white matter (here 36.1), $d_{csf}=$ water content of CSF (here 55.5), $N_{M}=$ number of metabolite protons contributing to the signal, and [W]= the concentration of water (here 55.5). Secondary analyses were also performed in which metabolites other than creatine were normalized to total creatine instead of water.

Spectral quality metrics of line width (FWHM), signal-to-noise ratio (SNR), and Cramer-Rao lower bound (CRLB) were calculated using the default algorithms in LCModel for PRESS and off-resonance MEGA-PRESS spectra. Recent *meta*-analytic evidence suggests some commonly used MRS quality thresholds (e.g., CRLB < 20 %, FWHM < 0.10 ppm) may be too liberal and thus obscure true metabolite abnormalities in people with schizophrenia due to excess nuisance variance from poorly estimated metabolite values (Smucny et al., 2021; Yang et al., 2023). Thus, we adopted inclusion criteria of FWHM ≤ 0.063 ppm and SNR ≥ 15 for all PRESS and MEGA-PRESS spectra, and we set CRLB ≤ 8 % as an inclusion threshold for LCModel fits of all PRESS-acquired metabolites except taurine. Note that CRLB is not calculated for GSH quantified by peak integration. Taurine partially overlaps other resonances and is difficult to measure reliably in humans at 3-Tesla without special sequences. However, taurine is more abundant in monkey than human brain, especially early in development (Sturman 1986), and it has been implicated in prior studies of MIA effects (Vernon et al., 2015). Thus, LCModel estimates of taurine were included for an exploratory analysis using a liberal CRLB threshold of ≤30 %. All spectra were visually examined for inclusion by an experienced MRS researcher (RJM) blinded to MIA status. Spectra with evident distortion or artifact were excluded, as were any values ≥3 SDs above or below the within-group mean at each age.

### 2.5. Behavioral testing

A detailed description of behavioral testing in this cohort and the effects of MIA on these behavioral measures has been previously described (Vlasova et al., 2021). In this prior publication, two behavioral measures showed the largest effect sizes for impairment in the MIA group. The largest effect size was for elevated omission errors following the first reversal of a simple object reversal learning task performed at 21 months of age in the Wisconsin General Testing Apparatus (WGTA) (Cohen’s d = 1.1) (Cohen, 1998). The omission errors were associated with behavior described as “apathetic/inactive” by blinded raters (Vlasova et al., 2021). The next largest effect size was for elevated omission errors during the simple discrimination reversal (SDR) phase of the Intradimensional/Extradimensional (ID/ED) task performed at 46–47 months of age administered via touchscreen computer using Cambridge Neuropsychological Test Automated Batteries software (CANTAB, Cambridge Cognition, Cambridge, UK). We reasoned that if abnormalities of prefrontal cortical metabolites were observed in the MIA group, it would be informative to examine possible associations between metabolite abnormalities and these behavioral impairments. This analysis could help determine whether the altered metabolites reflect a pathological process positively associated with behavioral impairment or instead reflect an adaptive process negatively associated with impairment and predict resilience in the face of MIA. Thus, associations were examined between altered metabolites and these two abnormal behavioral measures in the MIA group.

### 2.6. Statistical methods

Statistical analyses were conducted within a linear mixed-effects models framework (Laird and Ware, 1982) that can accommodate traditional general linear models (e.g., ANOVA and multiple linear regression) for data that were assumed normally distributed and independent across individuals, as well as linear mixed-effects models for normally distributed data that were collected for an individual across time. This flexible approach allows the use of all available data for an individual and provides the ability to control for the effect of covariates of interest and to account for the intrinsic complexity of the data by modeling subject-specific random effects and residual correlations. The covariance structures to model within-individual dependence were chosen based on the Bayesian information criterion. Transformations were employed if the assumptions of the models were not met.

To model brain metabolite measures from 6 to 45 months and assess whether MIA-treated animals had a different trajectory than the control animals, separate linear mixed-effects models were fitted for each brain metabolite measure using the maximum likelihood method. We first fitted models with fixed effects for group (MIA, control), age (6, 12, 24, 36, or 45 months), and the interaction between group and age. Within-individual dependence was modeled using spatial exponential correlation for NAA normalized by partial volume corrected water and GSH normalized by creatine, unstructured covariance for glutamate normalized by partial volume corrected water, and compound symmetry correlation for all other outcomes. If the interaction did not add significantly to the model, it was removed, and the results of the model including only main effects were reported. We then fitted a second set of models, which further adjusted for the tissue fraction of gray matter, using the same covariance structures except that GSH normalized by creatine used compound symmetry correlation. For both sets of models, significant group-by-age interactions were also examined and supported by Akaike information criterion and were followed up by planned comparisons between groups to determine which, if any, brain metabolite measures were significantly different between two groups at each of the five time points. The effect sizes were calculated using Cohen’s d = (MIA mean-Control mean)/SD (Cohen, 1998), where the group differences (MIA mean-Control mean) were estimated based on the fitted mixed-effects models, and SDs were the estimated standard deviations (after adjusting for fixed effects) from the fitted models.

To explore the association between the primary outcome (NAA normalized by partial volume corrected water) and two cognitive measures (number of omissions in the WGTA object reversal learning task at 21 months and miss rate at SDR stage in ID/ED test near 46–47 months) (Vlasova et al., 2021) within the MIA group, we used both Spearman’s correlation coefficients and linear mixed-effects models using the maximum likelihood method (Supplemental Methods). For the Spearman’s correlation, we first averaged the NAA over the 5 timepoints (6, 12, 24, 36, and 45 months) to obtain a summary NAA for each offspring who had complete NAA data. We then calculated the correlation between the summary NAA and each cognitive measure. For linear mixed-effects models, separate models were fitted for each cognitive measure, with NAA normalized by partial volume corrected water as the outcome. Models included fixed effects for age (6, 12, 24, 26, 45 months), the square-root transformed cognitive measure, and their interaction. For the cognitive measure of miss rate in ID/ED tests, the model further adjusted for age at the start of the ID/ED test. The spatial exponential correlation structure was used to account for within-animal dependence. The interactions were removed from the final models due to non-significance.

All models were validated both graphically and analytically. All tests were two-sided, with α = 0.05. All analyses were conducted in SAS version 9.4 (SAS Institute Inc., Cary, NC) and R 4.3.1 (R Foundation for Statistical Computing, Vienna, Austria).

## 3. Results

### 3.1. Maternal inflammatory response

As previously described in this same cohort of NHPs (Vlasova et al., 2021), MIA induced a maternal inflammatory response evidenced by clearly elevated serum IL-6 levels six hours following Poly IC injection. This was accompanied by fever and reduced appetite in the dams.

### 3.2. MRS measurement quality

Three of the 135 PRESS spectra were unusable due to acquisition errors, and three were excluded for distorted spectra. FWHM and SNR values for all remaining PRESS spectra, as calculated by LCModel, met our measurement quality inclusion criteria (all FWHM ≤ 0.048 ppm and all SNR ≥ 19). All CRLBs for all metabolites (except taurine) met our inclusion criterion (all ≤ 5 %). Coefficients of variation (COVs) for these water-scaled metabolite values across all animals at each age ranged from 3.9 % to 9.1 %, and no values were excluded as outliers. These metrics indicate excellent measurement quality and a low level of nuisance variance for these metabolites. Seven of the 129 taurine estimates had CRLB values above our relaxed criterion of ≤30 %. These data were excluded from analysis. COVs for taurine estimates across all animals at each age ranged from 10.3 % to 27.3 %, with higher COVs at older ages when taurine levels are lower. Representative PRESS spectra are shown in Fig. 1.

**Fig. 2.**
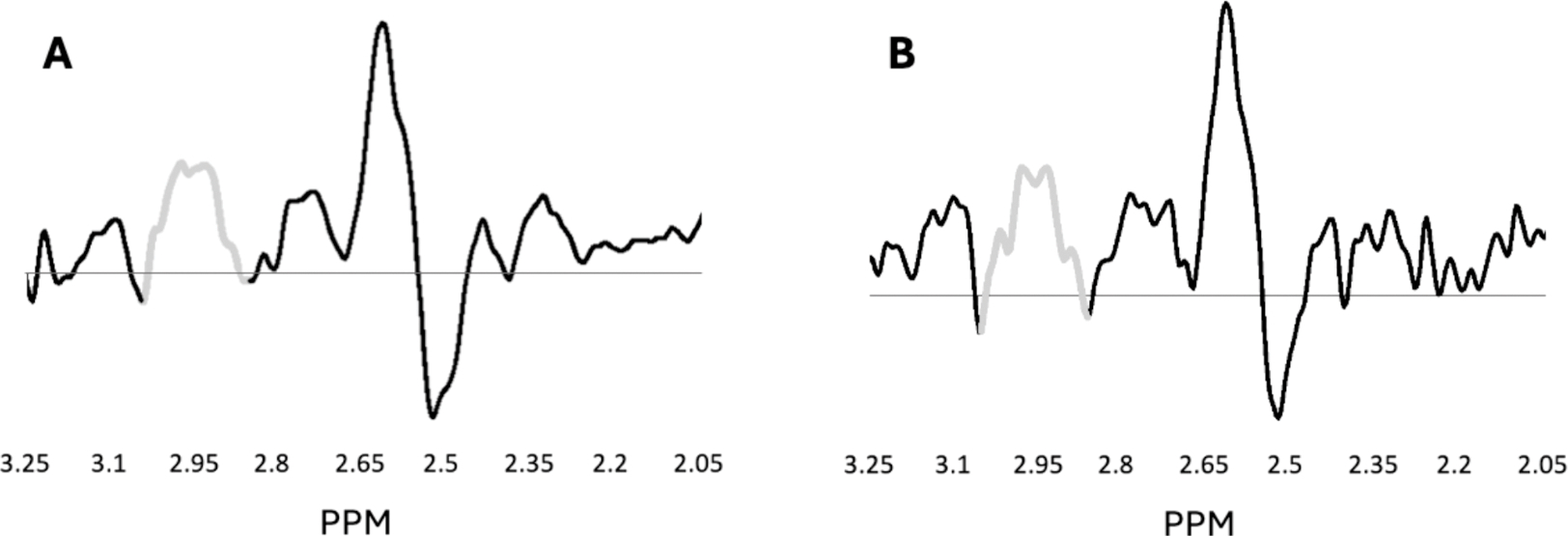
MEGA-PRESS difference spectra at 6 months (A) and 45 months of age (B). The edited glutathione cysteine resonance at 2.95 PPM is shown in gray. Co-editing resonances, including an NAA aspartate resonance at 2.67 PPM, are also visible. Panel A shows the average difference spectrum across all 6-month-old offspring (MIA and Control). Panel B shows an example difference spectrum from one 45-month-old MIA offspring.

Of the 135 MEGA-PRESS acquisitions, one was excluded for distortion. FWHM and SNR values for all MEGA-PRESS subspectra met our inclusion criteria (all FWHM ≤ 0.063 ppm and all SNR≥16). COVs for water-scaled GSH values across all animals at each age ranged from 19.8 % to 30.7 %. None were excluded as outliers. Representative MEGA-PRESS spectra are shown in Figs. 1 and 2.

### 3.3. MIA effects on prefrontal cortical metabolites

Summary prefrontal metabolite data for the MIA and control groups at each age are shown in Table 1. All seven metabolites showed significant age-related changes across the five measurements from 6 to 45 months. Table 2 summarizes the statistical results for group effects (MIA vs. control) and any age-specific group effects (if group-by-age interaction was significant) for all six metabolites included in the primary analysis and taurine in the exploratory analysis, adjusted by gray matter tissue fraction. Prefrontal NAA was significantly elevated in the MIA group across all ages (*est.* [estimated difference] = 0.38, *p* < 0.001). Prefrontal myo-inositol was significantly reduced in the MIA group across all ages (*est.* = − 0.29, *p* = 0.04). Creatine showed a significant group by age interaction (F=2.70, *p* = 0.034) with reduced creatine in the MIA group in the 36-month-old offspring (*est*. = − 0.29, *p* = 0.041). Taurine showed a significant group-by-age interaction (F=6.13, *p* < 0.001) with significantly elevated taurine levels in the MIA group at 36 and 45 months (*est.* = 0.27 and 0.26, *p* = 0.001 and 0.003, respectively) but not at earlier ages. No significant group or age-specific group effects were observed for glutathione, glutamate, or choline (see Table 2).

The same pattern of significant results was seen when metabolite values were not adjusted for tissue gray matter fraction, except that for creatine the 36-month group difference was not significant (Supplemental Table S1). Both elevated NAA and the age-specific elevation in taurine in the MIA group were also significant when metabolite estimates were normalized to creatine rather than tissue-corrected water. However, reduced myo-inositol in the MIA group became non-significant when normalizing by creatine (Supplemental Tables S2 and S3). The NAA data, both means and individual values at each age, are shown graphically in Supplemental Figures S1–S3, as are the myo-inositol and taurine data in Figures S4–S7.

### 3.4. Elevated NAA and behavioral performance in MIA animals

Since elevated NAA across all ages was the MIA-related brain metabolite abnormality with the largest effect size (Cohen’s *d* = 0.72), we examined its association with the two most consistent behavioral performance deficits observed in the MIA group (Vlasova et al., 2021). In MIA offspring, higher prefrontal NAA levels were associated with better performance on both measures. Higher prefrontal NAA levels were associated with fewer omission errors in the object reversal learning task at 21 months (*ρ* = −0.77, *p* = 0.04) and lower miss rates in the simple discrimination reversal touchscreen task at 46–47 months (*ρ*= − 0.78, *p* = 0.04). Results of the mixed-effects models confirmed these results (Table 3 and Supplemental Figures S8 and S9). Similar analyses for myo-inositol levels showed no significant relationship between reduced prefrontal myo-inositol and behavioral performance in the MIA offspring.

## 4. Discussion

In this first-ever MRS study of brain metabolites in an NHP model of MIA, we observed a significant elevation of prefrontal cortex levels of NAA in these male MIA offspring. This elevation was evident at the earliest measurement period and remained similarly elevated throughout the study from 6 to 45 months. Importantly, prefrontal NAA levels were inversely associated with measures of cognitive impairment in the 13 MIA animals, providing evidence that elevated NAA levels may be related to an adaptive, resilience-related process in response to MIA during the ages studied. Prefrontal myo-inositol levels were significantly reduced throughout the same period in the MIA animals but were not associated with measures of cognitive impairment. An exploratory analysis showed a significant elevation of prefrontal taurine levels in the MIA group at 36 and 45 months of age only. Prefrontal creatine levels were marginally reduced at 36 months in the MIA group. Prefrontal glutathione, glutamate, and choline levels showed no significant changes in the MIA animals.

### 4.1. Elevated prefrontal NAA

Based upon previous studies, there were reasons to expect a reduced level of prefrontal NAA rather than the elevated level observed from early childhood through adolescence in this cohort of male MIA offspring. Reduced NAA often reflects reduced neuronal integrity, and our prior reports demonstrated reduced frontal gray matter density and elevated cingulate cortex free water in this same cohort of NHPs across the same ages (Vlasova et al., 2021; Lesh et al., 2023). Furthermore, NAA levels are consistently reduced in a wide range of neurodevelopmental and neuropsychiatric conditions with hypothesized connections to neuroimmune disturbances, including children with autism (Du et al., 2023) and adults with schizophrenia (Yang et al., 2023), obsessive–compulsive disorder (Aoki et al., 2012), and HIV infection (Chelala et al., 2020). However, neither of the two prior MRS studies of Poly IC MIA observed reduced NAA in the offspring. Vernon et al. (2015) found that prefrontal NAA changed more from adolescence to adulthood in MIA rats than control animals, with non-significantly higher NAA in adolescence and non-significantly lower NAA in adulthood. In a study of adult offspring of MIA mice, Li et al. (2015) found significantly elevated NAA in the anterior cingulate cortex. This latter result is consistent with the current finding of elevated prefrontal NAA in MIA NHPs.

Converging clinical and preclinical evidence suggests that MIA acts as a neurodevelopmental disease primer, which is relevant to a range of neurodevelopmental and neuropsychiatric conditions (Meyer, 2014). However, there is significant resilience to the phenotypic manifestations of MIA in both human epidemiological studies and animal models (Meyer, 2019; Estes et al., 2020). The range of possible neurobiological effects of MIA will likely include both pathogenic processes and homeostatic adaptations with protective effects, which may determine neurodevelopmental outcomes. The current finding of elevated prefrontal NAA associated with relatively spared cognition suggests that increased NAA levels may represent one element of a beneficially adaptive or resilience-related process in the MIA NHPs, at least during the developmental stages studied here.

A better understanding of the regulatory pathways leading to this increase in NAA levels could help elucidate possible mechanisms of such putative beneficial adaptations, which could have clinical relevance for children exposed to MIA. Elevated dopaminergic activity has been associated with increased NAA levels under some conditions and is a potential mechanism of the elevated NAA observed in the MIA animals here. The biosynthetic enzyme for NAA in the brain is aspartate N-acetyltransferase (Ariyannur et al., 2010). Studies by Nitta and colleagues in mice have shown that increased dopaminergic activity due to six days of methamphetamine administration strongly upregulated aspartate N-acetyltransferase expression in the medium spiny neurons of the nucleus accumbens (Niwa et al., 2007), which in turn was associated with increased NAA synthesis in this ventral striatal structure (Miyamoto et al., 2014). Perrine et al., (2010) observed elevated PFC NAA in rats seven days after one day of repeated 3,4-methylenedioxymethamphetamine (MDMA) administration. However, Meyer et al. (2006) saw no effect on frontal NAA levels in marmoset monkeys one week after a similar MDMA exposure. In contrast, chronic exposure to stimulant drugs is associated with reduced prefrontal NAA in humans (Smucny and Maddock, 2023). However, this may be due to neurotoxic rather than neuromodulatory processes (Fonseca et al., 2017). Prior PET studies from our group have shown increased striatal presynaptic dopamine in the offspring of MIA monkeys, including in the current cohort (Bauman et al., 2019; Smucny et al., 2023). Unfortunately, dopamine levels in the prefrontal cortex could not be measured with the PET method used for these studies. However, Winter et al. (2009) observed elevated prefrontal cortex dopamine levels in the adult offspring of early-middle gestation MIA mice using high-performance liquid chromatography on post-mortem tissue. If dopamine is also elevated in the prefrontal cortex in the current MIA monkeys during childhood and adolescence, it could contribute to the elevated NAA seen here.

Altered regulatory pathways involving cytokines and neurotrophic factors are also potential mechanisms for a sustained increase in prefrontal NAA levels in MIA animals. Erythropoietin (epo) is best known for its synthesis by the kidney and its capacity to promote the proliferation of red blood cell progenitors. However, it is also synthesized within the brain by astrocytes and, to a lesser extent, by neurons (Simões et al., 2018). Epo is a glycoprotein hormone with cytokine properties in the brain, including both anti-inflammatory and neuroprotective effects (Alnaeeli et al., 2012). Simões et al. (2018) recently showed that MIA induced by lipopolysaccharide in rats triggered elevated epo levels in fetal brain tissue, and Singhal et al. (2018) demonstrated that exogenously administered epo evokes increased NAA in the cortical gray matter of mice. Sickle cell anemia is a chronic condition associated with elevated epo levels and elevated brain NAA levels (Steen and Ogg, 2005), and mouse models of sickle cell disease also show elevated levels of brain NAA (Cui et al., 2017). These studies of epo effects are an example of how altered regulation of cytokines and other signaling molecules could influence brain NAA levels. Interestingly, NAA participates in the regulation of brain-derived neurotrophic factor (BDNF) expression, such that higher levels of NAA lead to higher BDNF levels (Francis et al., 2006). Several studies have reported reduced brain levels of BDNF in the prefrontal cortex or hippocampus of MIA offspring in rodent models (Han et al., 2016; Talukdar et al., 2020; Abu-Ata et al., 2023). This suggests a possible scenario in which low BDNF in the current NHP MIA offspring could have triggered a regulatory increase in NAA in service of raising BDNF back toward normal levels. Overall, these findings suggest the hypothesis that altered activity in one or more neuromodulatory processes may contribute to elevated prefrontal NAA and its apparent association with preserved cognitive function in the MIA NHPs. Future investigations of NAA, cognition, and candidate neuromodulators in MIA offspring are needed to examine this hypothesis further.

### 4.2. Reduced prefrontal myo-inositol

Prefrontal myo-inositol levels were reduced across all ages in the MIA animals. This agrees with the finding of Li et al. (2015), who found reduced anterior cingulate myo-inositol in MIA mice but differs from the report of Vernon et al. (2015), where no effect on prefrontal myoinositol in MIA rats was noted. Our findings are similar to the reduced myo-inositol in frontal regions that have been reported in MRS *meta*-analyses of patients with schizophrenia (Das et al., 2018) and autism (Aoki et al., 2012), both of which are neurodevelopmental disorders that have been associated with maternal immune activation. Myo-inositol has a major role as a non-perturbing osmolyte present in all brain cell types (Fisher et al., 2002; Rae, 2014). As such, reduced brain myoinositol is consistently observed in disturbances of water balance in the direction of hypo-osmolality or hyponatremia (Restuccia et al., 2004; Sterns and Silver, 2006). We recently reported significantly increased extracellular free water in cingulate cortex gray matter in this same cohort of MIA NHPs (Lesh et al., 2023). This abnormality may entail an extracellular hypo-osmolality, which could lead to reduced prefrontal myo-inositol in the MIA group. However, an exploratory correlational analysis showed no association between prefrontal free water and myo-inositol levels in the MIA group. In contrast to the current findings, elevated brain myo-inositol has been reported in patients with HIV infection (Chelala et al., 2020) and in animal models of reactive astrocytes and astrogliosis (Kim et al., 2005; Ligneul et al., 2019). Overall, the finding of reduced prefrontal myo-inositol in the NHP MIA offspring is similar to prior reports in conditions associated with abnormal neurodevelopment and unlike the elevated myo-inositol that has been associated with ongoing neuroinflammation.

### 4.3. Late increase in prefrontal taurine

Our exploratory analysis of taurine showed that prefrontal taurine levels were elevated at 36 and 45 months in the MIA group. Taurine is a widely distributed amino acid in the brain with antioxidant, anti-inflammatory, and osmoregulatory actions, and it influences many neural processes, including inhibitory and excitatory neurotransmission (Huxtable, 1989; Jakaria et al., 2019). Elevated prefrontal taurine in these adolescent Poly IC MIA animals contrasts with earlier reports that anterior cingulate cortex taurine is unchanged in adolescent and reduced in adult offspring of MIA rats (Vernon et al., 2015) and unchanged in the prefrontal cortex but reduced in the hippocampus of adult MIA mice (Winter et al., 2009). Since the CRLB quality metric values for the taurine measurements were much worse than those for other PRESS-acquired metabolites and exceeded conventional quality thresholds, the current finding of elevated prefrontal taurine in the adolescent MIA NHPs should be considered preliminary until confirmed by future studies.

### 4.4. Unchanged prefrontal metabolites

We observed no significant changes in prefrontal GSH levels in the MIA group at any of the childhood or adolescent ages studied here. This result is consistent with the only other report of MRS measures of GSH in an MIA model. Vernon et al. (2015) found no change in prefrontal GSH in MIA rats at adolescent ages, although they reported reduced GSH in adult MIA rats. In the current study, coefficients of variation (COVs) for GSH measurements at each age in each group were substantially higher than those for other metabolites (except for taurine), with a median value of 26%. The relatively high COVs suggest appreciable noise variance in the GSH measurements, which could have obscured an underlying true difference between the groups. Noise variance can be reduced by averaging over repeated measurements for each subject. Table 1 shows that GSH levels in both MIA and Control groups were similar at ages 6, 12, and 24 months and higher at 36 and 45 months. Based on this pattern, we averaged the GSH values at 6, 12, and 24 months and separately averaged the values at 36 and 45 months. The resulting COVs were lower (median value of 18%). Reanalyzing the GSH data using only these two, less noisy, averaged values confirmed the original finding that MIA is not associated with a group difference in prefrontal GSH levels during childhood and adolescence. Studies in adult offspring would be necessary to determine if GSH levels are abnormal in adulthood in MIA NHPs.

No significant changes were observed in our primary analyses of glutamate, choline, and creatine, except that reduced creatine at 36 months in the MIA group was marginally significant. In agreement with the earlier rodent MIA studies (Vernon et al., 2015; Li et al., 2015), changes in these three metabolites are not prominent consequences of MIA in this NHP model system.

### 4.5. Limitations

There are several limitations to the inferences that can be drawn from this study. Most importantly, our sample of NHPs includes only male animals. There is growing evidence that sex may have a significant influence on MIA effects (Coiro and Pollak, 2019; Estes et al. 2020). The current studies were initiated before the NIH policy requiring sex as a biological variable to be factored into experimental design. As such, understanding sex differences in the NHP model lags behind the more rapid progress in the rodent MIA model (Bergdolt and Dunaevsky, 2019; Estes et al. 2020). Our ongoing studies using a larger cohort that includes female animals will allow us to examine possible sex differences in the neurometabolic characteristics of this NHP MIA model. Another limitation is sample size. While robust for an NHP gestational model, the sample size of N = 14/group was relatively modest, which limited our ability to detect any more subtle group differences in brain metabolites. Given the small sample size, the correlations between NAA levels and cognitive performance will be more compelling with replication. In quantifying metabolite values with partial volume corrected water normalization, only canonical values derived from adult humans for the water content of gray and white matter were available for the calculations (Near et al., 2021), and the implicit assumption that these values do not change during development is almost certainly untrue. Our water correction equation was also incomplete as it lacked corrections for relaxation times, the values of which are unknown for developing macaques. However, the pattern of results was very similar for water-normalized and creatine-normalized values, suggesting that nuisance variance related to the water estimates is not driving the results.

### 4.6. Conclusions

Prior studies support the construct validity of the first-trimester exposure NHP MIA model for studying the pathophysiology of abnormal neurodevelopment in the context of maternal infection (Vlasova et al. 2021; Lesh et al., 2023). The current study adds a neurometabolic perspective to the growing understanding of this model. The male MIA offspring had significantly elevated prefrontal NAA throughout their development from 6 to 45 months of age. The positive association between NAA and performance on cognitive tests sensitive to the subtle impairment observed in the MIA group suggests that elevated prefrontal NAA may represent an adaptive response associated with preserved cognition in the MIA offspring during childhood and adolescence. Planned analyses of brain tissue from this cohort may provide valuable insights into the cellular and molecular mechanisms and the pathophysiological significance of the brain abnormalities previously observed *in vivo* (Vlasova et al., 2021; Lesh et al., 2023; Smucny et al., 2023), including the neurometabolic changes reported here.

## Supplementary Material

Supplemental Materials

Supplemental Materials2

## Figures and Tables

**Table 1:** Summary statistics for prefrontal brain metabolite measures.

	6 Months		12 Months		24 Months		36 Months		45 Months	
	MIA^1^	Control	MIA^2^	Control	MIA^3^	Control^4^	MIA^3^	Control^3^	MIA^3^	Control^3^
	(*n* = 14)	(*n* = 13)	(*n* = 13)	(*n* = 14)	(*n* = 13)	(*n* = 14)	(*n* = 13)	(*n* = 14)	(*n* = 12)	(*n* = 14)

Age (days), *mean* (*SD*) [*Range*]										
	179.9 (1.6)	181.2 (5.5)	365.5 (1.5)	365.1 (0.7)	730.1 (1.3)	729.8 (1.0)	1094.6 (1.0)	1098.9 (8.2)	1371.6 (1.9)	1370.9 (1.6)
	[177–182]	[177--199]	[364–369]	[364–366]	[729–733]	[729–732]	[1093–1096]	[1092–1116]	[1368–1374]	[1368–1374]
Prefrontal metabolites measures (normalized by partial volume corrected water, unadjusted for gray matter tissue fraction), *mean* (*SD*)										
NAA	9.3 (0.7)	8.8 (0.7)	7.9 (0.6)	7.5 (0.6)	7.9 (0.8)	7.4 (0.5)	8.0 (0.4)	7.7 (0.3)	8.1 (0.3)	8.0 (0.3)
Inositol	7.3 (0.6)	7.6 (0.3)	6.3 (0.5)	6.6 (0.6)	6.1 (0.6)	6.3 (0.3)	5.9 (0.7)	6.3 (0.3)	5.7 (0.5)	6.0 (0.4)
Glutamate	13.7 (0.9)	13.3 (0.9)	11.1 (0.6)	11.2 (0.9)	10.2 (0.7)	10.1 (0.7)	10.1 (0.7)	10.1 (0.5)	10.0 (0.5)	10.0 (0.5)
Choline	1.7 (0.1)	1.6 (0.2)	1.4 (0.1)	1.4 (0.1)	1.4 (0.1)	1.4 (0.1)	1.4 (0.1)	1.4 (0.1)	1.3 (0.1)	1.4 (0.1)
Creatine	8.3 (0.3)	8.4 (0.4)	7.7 (0.4)	7.6 (0.4)	7.4 (0.4)	7.3 (0.4)	7.3 (0.4)	7.6 (0.3)	7.2 (0.5)	7.5 (0.4)
Taurine	2.3 (0.3)	2.5 (0.2)	1.5 (0.3)	1.4 (0.3)	1.2 (0.3)	1.0 (0.1)	1.3 (0.3)	1.0 (0.2)	1.1 (0.2)	0.9 (0.2)
GSH^5^	2249 (691)	2260 (615)	2097 (414)	2137 (546)	1990 (571)	2063 (420)	2484 (633)	2563 (509)	2598 (740)	2646 (524)

Note: MIA = maternal immune activation, SD = standard deviation, NAA = n-acetylaspartate, GSH = glutathione.

1 Three values were missing for all metabolites except GSH.

2 Two were missing for all metabolites except GSH.

3 Taurine value was missing for 1 animal.

4 Taurine values were missing for 2 animals.

5 Institutional units for GSH (acquired with MEGA-PRESS) are scaled differently than all other metabolites (acquired with PRESS).

**Table 2:** Group differences in prefrontal brain metabolites normalized by partial volume corrected water from the linear mixed-effects models, adjusted by gray matter tissue fraction.

Estimated difference between MIA and Control^1^												
	Overall group difference		Age-specific group difference									
			6 Months		12 Months		24 Months		36 Months		45 Months	
Outcome	*Estimate (SE)*	*P-value*	*Estimate (SE)*	*P*-value	*Estimate (SE)*	*P*-value	*Estimate (SE)*	*P*-value	*Estimate (SE)*	*P*-value	*Estimate (SE)*	*P*-value

NAA	**0.38 (0.10)**	**0<0.001**	-	-	-	-	-	-	-	-	-	-
Inositol	**−0.29 (0.13)**	**0.03**	-	-	-	-	-	-	-	-	-	-
Glutamate	0.19 (0.12)	0.12	-	-	-	-	-	-	-	-	-	-
Choline	0.01 (0.04)	0.73	-	-	-	-	-	-	-	-	-	-
Creatine	-	-	−0.04 (0.14)	0.80	0.07 (0.14)	0.60	0.05 (0.14)	0.72	**−0.29 (0.14)**	**0.04**	−0.20 (0.14)	0.17
Taurine	-	-	−0.10 (0.08)	0.25	0.06 (0.08)	0.45	0.10 (0.08)	0.22	**0.27 (0.08)**	**0.001**	**0.26 (0.08)**	**0.003**
GSH	−41.0 (121.2)	0.74	-	-	-	-	-	-	-	-	-	-

Abbreviations: MIA=maternal immune activation, SE=standard error, NAA=n-acetylaspartate, GSH=glutathione.

1 Estimated differences and P-values for brain metabolites measures from linear mixed-effects models that included fixed effects for group (MIA, Control), age at testing (6, 12, 24, 26, 45 months), their interaction, and tissue fraction gray matter, with covariance structure to account for within-animal correlation (spatial exponential correlation for NAA, unstructured covariance for glutamate, and compound symmetry correlation for all other metabolites). If interaction of group with age was significant, estimated age-specific group differences were reported. If the interaction of group with age was not significant, it was removed from the final model; thus, the estimated group difference was the same across all ages (hence only the overall group difference was reported).

**Table 3:** Associations between prefrontal NAA (normalized by partial volume corrected water) and cognitive measures in MIA group.

	Spearman’s Rank Correlation^1^ Coefficient^1^		Estimated Coefficient from Linear Mixed-effects Models^2^	
Cognitive Measures	*Correlation*	*P*-value	*Estimate (SE)*	*P*-value

Number of omissions in Reversal Learning task at 21 months	**−0.77**	**0.04**	**−0.19 (0.07)**	**0.01**
Miss rate at SDR stage in ID/ED test near 46–47 months	**−0.78**	**0.04**	**−0.85 (0.32)**	**0.02**

Abbreviations: MIA=maternal immune activation, SE=standard error, Reversal Learning = simple object reversal from Wisconsin General Testing Apparatus, SDR=simple discrimination reversal, ID/ED=Intradimensional/Extradimensional shift test, NAA=n-acetylaspartate.

1 Spearman’s rank correlation coefficients and P-values between NAA (averaged over 5 timepoints, i.e., 6, 12, 24, 36, 45 months) and the two cognitive measures showing greatest impairment in MIA animals. Animals with missing NAA values at any time point were excluded, leading to a total of 7 animals in the MIA group included in the analysis.

2 Estimated coefficients for the two cognitive measures from linear mixed-effects models (separate model for each cognitive measure), using 14 MIA animals (3 with MRS data missing at 6 months, 2 with MRS data missing at 12 months, 1 missing at 45 months due to death, and 1 missing since 12 months due to death). The outcome variable is NAA at 6, 12, 24, 36, 45 months. The fixed effects included the age of measuring NAA (6, 12, 24, 26, 45 months), the square-root transformed cognitive measure, and their interaction, with spatial exponential correlation structure to account for within-animal correlation. For the cognitive measure of miss rate in ID/ED tests, the model further adjusted for the age at the start of ID/ED test. The interactions were removed from the final models due to non-significance. The omission number and miss rates were square-root transformed, and the corresponding estimated coefficients can be interpreted as the average change in NAA for a 1-unit increase in square-rooted omission number or miss rates.

## Data Availability

Data will be made available on request.
