# Supplementary material for: Altered brain metabolites in male nonhuman primate offspring exposed to maternal immune activation
Source: Brain Behav Immun. Author manuscript; Available in PMC 2025 Feb 10. (PMC11809764; doi:10.1016/j.bbi.2024.07.011)
Supplement: Supplemental Materials [file NIHMS2052597-supplement-Supplemental_Materials.pdf]

Maternal Immune Activation Model Reporting Guidelines Checklist

ARRIVE Reporting Guideline & Recommendation	Arrive Item	MIA Model Specific Reporting Recommendation<br>*Please complete this chart for each point outlined below. If not applicable, write N/A*
**Study design**<br>➢Overview of immune activation issues<br><br>For each experiment, give brief details of the study design including:<br>a. The number of experimental and control groups.<br>b. Any steps taken to minimize the effects of subjective bias when allocating animals to treatment (e.g. randomization procedure) and when assessing results (e.g. if done, describe who was blinded and when).<br>c. The experimental unit (e.g. a single animal, group or cage of animals).<br><br>A time-line diagram or flow chart can be useful to illustrate how complex study designs were carried out.	6	MIA Specific Reporting:<br>a. General need for improved reporting in MIA model methods + reporting pilot data<br>○ Details on pilot data:<br><br>The MIA-induction protocols are based on our previous dosing (Weir et al., 2015) and gestational time line comparisons (Bauman et al., 2014). For the present study, the effectiveness of Poly ICLC to induce an immune response was piloted in a single non-pregnant female rhesus macaque. Immune response was measured by change in cytokine levels, specifically IL-6, in blood samples taken prior to Poly ICLC injection and 6 hours after injection. Temperature was also monitored pre-and post-injection.
**Experimental procedures**<br>➢Compounds<br>➢Validation measures<br><br>For each experiment and each experimental group, including controls, provide precise details of all procedures carried out. For example:<br>a. How (e.g. drug formulation and dose, site and route of administration, anaesthesia and analgesia used [including monitoring], surgical procedure, method of euthanasia). Provide details of any specialist equipment used, including supplier(s).<br>b. When (e.g. time of day).<br>c. Where (e.g. home cage, laboratory, water maze).<br>d. Why (e.g. rationale for choice of specific anaesthetic, route of administration, drug dose used).	7	Provide details of:<br>a. Compounds – source, vehicle, preparation/storage, administration route, volume administered, whether anesthetics were used at time of immune challenge.<br>○ Name of compound: Poly ICLC<br>○ Catalogue number: N/A<br>○ Lot number: N/A<br>○ Vehicle control used: Sterile saline<br>○ Route of administration: IV<br>○ Volume administered: ~1 ml (dose: 0.25 mg/kg, concentration: 2 mg/mL)<br>○ Storage conditions: Refrigerated<br>○ Anesthetic (type, dose, duration) used:<br>N/A<br>b. Housing variables at injection - temperature of room at injection time, cage change at time of injection or not<br>○ Light cycle of animal housing room: Lights on 0600-1800<br>○ Time of day of injection: 7:30AM<br>○ Room temperature at injection time: 18-29 C<br>○ Did a cage change occur at time of injection: NO

Kentner AC, Bilbo AD, Brown AS, Hsiao EY, McAllister AK, Meyer U, Pearce BD, Pletnikov MV, Yolken RH, Bauman MD. (2018). Maternal immune activation: reporting guidelines to improve the rigor, reproducibility, and transparency of the model. Neuropsychopharmacology, https://doi.org/10.1038/s41386-018-0185-7.

		c. Validation of immune activation – behavior, physiological indices and/or cytokine data, including pilot dosing data<br>○ Method used to verify immune activation:<br>Cytokine levels were evaluated from pre-dosing baseline blood samples, 6 hours after injection #2, 6 hours after injection #3, and post-dosing baseline blood samples. Temperature and sickness behaviors were monitored pre-dosing, throughout the dosing period, and post-dosing.<br>d. Validation of gestational timing – vaginal plug, estrous cycle, weight gain<br>○ Method of validating gestational timing:<br>Dams were from the time-mate colony at the CNPRC. Breeding was based on menses cycle, and pregnancies were confirmed and dated via ultrasound.<br>Additional comments:<br>Magnetic resonance images were collected at 6, 12, 24, 36, and 45 months of age using a Siemens Magnetom Skyra 3-T (Davis, California) with an 8-channel coil optimized for nonhuman primate brain scanning (RapidMR, Columbus, Ohio). While twenty-four of the animals were also scanned at one month of age (and three animals at three months), poor image quality and low gray/white T1
**Experimental animals**<br>➢Species/strain/vendor<br>a. Provide details of the animals used, including species, strain, sex, developmental stage (e.g. mean or median age plus age range) and weight (e.g. mean or median weight plus weight range).<br>b. Provide further relevant information such as the source of animals, international strain nomenclature, genetic modification status (e.g. knock-out or transgenic), genotype, health/immune status, drug or test naïve, previous procedures, etc.	8	Provide details of:<br>a. Species – considerations for appropriate species (mouse, rat, non human primate, other)<br>○ Species: Macaca mulatta<br>b. Strain – variability in strain can influence model<br>○ Strain: N/A<br>c. Maternal/Offspring Physiological Variables at time of immune challenge – age, body weight<br>○ Maternal Age at challenge: 5-13 years<br>○ Maternal Body weight: 5-10 kg<br>○ Offspring Age at challenge: N/A<br>○ Offspring Sex: Males only tested<br>○ Offspring Body weight:<br>d. Vendor – even within the same strain, vendor can influence endpoints<br>○ Vendor: CNPRC breeding colony<br>○ Location of Vendor:<br>○ Room/area where animals originated from:

		Additional Comments:
**Housing and husbandry**<br>➢Cage, ventilation, bedding, enrichment<br><br>Provide details of:<br>a. Housing (type of facility e.g. specific pathogen free [SPF]; type of cage or housing; bedding material; number of cage companions; tank shape and material etc. for fish).<br>b. Husbandry conditions (e.g. breeding program, light/dark cycle, temperature, quality of water etc for fish, type of food, access to food and water, environmental enrichment).<br>c. Welfare-related assessments and interventions that were carried out prior to, during, or after the experiment.	9	Provide details of:<br>a. Caging systems<br>○ *At breeding*<br>Material of cage: Stainless steel<br>Cage dimensions: 60 x 65 x 79 cm<br>○ *After parturition*<br>Material of cage: Stainless steel<br>Cage dimensions: 60 x 65 x 79 cm<br>○ *At weaning*<br>Material of cage: Stainless steel<br>Cage dimensions: 60 x 65 x 79 cm<br><br>b. Animal Holding room<br>○ Temperature in room: 18-29 C<br>○ Humidity in room: 30-70%<br>○ Ventilation system: 10-15 air exchanges/hour<br>○ Specific pathogen free [SPF]: NO<br>○ Are males & females housed in the same or separate rooms: Housed in same room after weaning<br>c. Bedding exchanges/bedding type<br>○ Type of cage bedding used: N/A<br>○ Frequency of cage changes per week<br>*during gestation:* Daily sanitation; cage change every two weeks<br>*during neonatal period:* Daily sanitation; cage change every two wee<br>*following weaning:* Daily sanitation; cage change every two weeks<br><br>d. Breeding - bred on site or timed pregnant, how many different sires (are the same fathers breeding with both experimental and control dams)<br>Breeding location: CNPRC

- Gestational age at shipping: N/A
- Biological age of dams (if not listed in Section 8c): 5-13
- Number of Dams bred: see below
- How many times have dams been mated previously: 0-7
- How many times did the dams mate and not become pregnant:
- Are the dams primiparous or multiparous? Dams are a mix of primi- & mult
- What was the frequency of maternal handling during the gestational/neonatal period (e.g. cage cleanings, weighing, blood collection manipulations): I ultrasound and blood draw/trimester
- Biological age of sires: see below
- Number of sires bred: see below
- How many times have sires been mated previously: see below
- How many times did the sires mate successfully (e.g. mating resulted in pregnancy, full term birth): see below
- If bred previously, what was the interval between mating times:
- Are sires matched to experimental and control dams: NO
- Describe the mating design (1:1, 1:2 etc): 1:1

e. Social enrichment – number of cage companions
  - Number of cage companions prior to breeding: 0-1
  - Gestational age when dam separated for parturition: N/A
  - Number of cage companions at weaning: 1

f. Physical enrichment – describe enrichment devices, and when enrichment is in the cage (removed when pups born? Or present throughout study), does the enrichment type change? How frequently?
  - Describe what type of enrichment devices (and how many) are included in cage/housing room:

Standard CNPRC enrichment was provided throughout the study. This included mirrors, Kong toys and other physical manipulanda, foraging boards, rotational hanging and foraging toys, coconuts, videos, and rotations in larger enclosures.

		○ Does enrichment type/access change across study? NO<br>○ If so, when does enrichment type/access change (e.g. enrichment removed prior to parturition and replaced in late neonatal period):<br><br>Additional Comments:<br><br>Social enrichment: Infants were raised in individual cages with their mothers where they had visual access to other mother-infant pairs at all times. For 3 hours each day, one familiar adult male and four familiar mother-infant pairs were allowed to freely interact in a large cage (3m l x 1.8m w x 2m h) to provide enrichment and facilitate species-typical social development. The infants were weaned from their mothers at 6 months of age and were permanently paired with a familiar peer from their rearing group. Weanlings continued the same socialization routine through approximately 18 months of age. They were transferred to the large enclosures for 3 hours each day with the same three weanlings from their rearing group, the familiar adult male, and an adult female.
**Sample size**<br>➢ Litter versus offspring<br><br>a. Specify the total number of animals used in each experiment, and the number of animals in each experimental group.<br>b. Explain how the number of animals was arrived at. Provide details of any sample size calculation used.<br>c. Indicate the number of independent replications of each experiment, if relevant.	10	Provide details of:<br>a. Maternal N vs offspring N<br>○ What is the total number of dams/litters included in the study: N=28<br>○ What is the total number of offspring per litter included the study: N=28 offpsring<br>b. Litter size and sex distribution<br>○ What size was each litter maintained at:<br>○ What age did culling take place at:<br>○ How many males and females were maintained in each litter:<br><br>c. Cross fostering<br>○ Did cross fostering occur: NO<br>○ If so, at what age did cross fostering occur:<br><br>Additional Comments:

**Allocating animals to experimental groups**<br><br>a. Give full details of how animals were allocated to experimental groups, including randomization or matching if done.<br>b. Describe the order in which the animals in the different experimental groups were treated and assessed.	11	a. How many offspring per litter were used in each measure:<br><br>b. Randomization/Matching procedures<br>○ What procedures were used to assign animals to groups:<br>Dam assignment to experimental groups was balanced by age, weight, and prior conception number. Groups were filled in an alternating pattern as much as possible while taking into account the above factors.<br><br>c. Sex as a biological variable (behavioral and physiological outcomes)<br>○ Were both males and females evaluated in each behavioral and physiological outcome: NO<br><br>Additional Comments:
**Experimental outcomes**<br>➢ Behavioral testing<br>➢ Physiological endpoints<br><br>Clearly define the primary and secondary experimental outcomes assessed (e.g. cell death, molecular markers, behavioral changes).	12	a. Maternal behavior and pup interactions<br>○ If maternal care was evaluated, were there differences following immunogen challenge (if so, please briefly describe):<br>N/A<br><br>b. Age(s) of offspring at behavioral testing/physiological evaluation endpoints:<br>0-4 years of age<br><br>c. Order of testing (e.g. behavioral test order)<br>○ Were animals evaluated in a counter-balanced order in terms of:<br>*presentation of tests to each animal:* NO<br>*order of experimental/control groups run through each test:* YES<br>○ What was the inter-test interval if a single animal underwent a battery of tests:<br>N/A

		Additional Comments:<br>Offspring participated in a comprehensive evaluation of brain and behavioral development from birth - four years. Behavioral data included neonatal assessments (1wk), visual paired comparison (1mos), 24 hour biobehavioral assessment (3mos), home cage and social cage observations (0-18mos), noninvasive social eye-tracking, novel cage and novel social partner observations (11, 23, 35mos), WGTA reversal learning (18mos), CANTAB (32-47mos). Magnetic resonance imaging was performed at approximately 6, 12, 24, 36, and 45 months of age and positron emission tomography (PET) imaging at 14, 26, 38, and 48 months of age. Biological samples (blood, cerebrospinal fluid, hair, feces) were collected periodically.
**Statistical methods**<br><br>a. Provide details of the statistical methods used for each analysis.<br>b. Specify the unit of analysis for each dataset (e.g. single animal, group of animals, single neuron).<br>c. Describe any methods used to assess whether the data met the assumptions of the statistical approach.	13	a. Unit of analysis for each data set<br>○ Is the unit (n) of each analysis based on number of litters, or number of animals used per group:<br>N=14 MIA-treated offspring (1 missing at 45 months and 1 missing after 6 months due to death) were compared to N=14 control offspring (litter considerations do not apply). Statistical analyses employed linear mixed-effects models for longitudinal data which account for the correlated nature of the data.
**Other Disclosures**		Please make note of any other extraneous variables that you would like to report (e.g. fire alarms, construction, temporary relocations, other variables that you think we should be considering in our studies etc.):<br>We have adapted the MIA reporting guidelines generated for rodent MIA models for use in nonhuman primates. Additional methodological details are available upon request.

The recommended use of this reporting form is to fill it out and include it as supplemental material for each of your laboratory's research publications. If there are difficulties utilizing/adapting this fillable form, please contact one of the corresponding authors to request a copy. The authors give permission for this table to be edited for use in reporting on other animal models (e.g. postnatal immune challenge models, early life stress models) as appropriate.

Kentner AC, Bilbo AD, Brown AS, Hsiao EY, McAllister AK, Meyer U, Pearce BD, Pletnikov MV, Yolken RH, Bauman MD. (2018). Maternal immune activation: reporting guidelines to improve the rigor, reproducibility, and transparency of the model. Neuropsychopharmacology, https://doi.org/10.1038/s41386-018-0185-7.
