# Supplementary material for: Altered brain metabolites in male nonhuman primate offspring exposed to maternal immune activation
Source: Brain Behav Immun. Author manuscript; Available in PMC 2025 Feb 10. (PMC11809764; doi:10.1016/j.bbi.2024.07.011)
Supplement: Supplemental Materials2 [file NIHMS2052597-supplement-Supplemental_Materials2.pdf]

Altered Brain Metabolites in Male Nonhuman Primate Offspring Exposed to Maternal Immune Activation

Richard J. Maddock, Roza M. Vlasova, Shuai Chen, Ana-Maria Iosif, Jeffrey Bennett, Costin Tanase, Amy M. Ryan, Takeshi Murai, Casey E. Hogrefe, Cynthia M. Schumann, Daniel H. Geschwind, Judy Van de Water, David G. Amaral, Tyler A. Lesh, Martin A. Styner, A. Kimberley McAllister, Cameron S. Carter, Melissa D. Bauman

Supplemental Materials

Supplemental Methods: Rationale for the approach to testing associations between NAA and cognitive performance

Table S1. Estimated differences and standard errors from the linear mixed-effects models assessing the relationship between group and prefrontal brain metabolites measures, not adjusted by gray matter tissue fraction.

Table S2. Summary for creatine-normalized prefrontal brain metabolite measures.

Table S3. Group differences in creatine-normalized prefrontal brain metabolites from the linear mixed-effects models, adjusted by gray matter tissue fraction.

Figure S1. Prefrontal NAA normalized to partial volume corrected water

Figure S2. Prefrontal NAA normalized to creatine

Figure S3. Individual NAA values at each age for each animal

Figure S4. Prefrontal Myo-inositol normalized to partial volume corrected water

Figure S5. Individual Myo-inositol values at each age for each animal

Figure S6. Prefrontal Taurine normalized to partial volume corrected water

Figure S7. Individual Taurine values at each age for each animal

Figure S8. Inverse association between NAA and reversal learning at age 21 months in MIA group

Figure S9. Inverse association between NAA and miss rate on simple discrimination learning task at age 46-47 months in MIA group

Supplemental Methods: Rationale for the approach to testing associations between NAA and cognitive performance

Each of the two cognitive measures showing impairment were measured only once, one at age 21 months and one at age 46-47 months. In contrast, prefrontal NAA was measured repeatedly, and it was elevated across all five ages with no evidence of a group by age interaction. In testing for associations between elevated NAA and cognitive performance, we had a choice of using either NAA levels measured at the (approximately) same time as the cognitive data or using all NAA levels acquired from 6 to 45 months of age. Importantly, the age-specific NAA values have much higher coefficients of variation than the NAA values averaged across all ages. In testing the correlation between NAA and cognitive performance, this extra variance could be informative or it could be nuisance variance, depending on the nature of the association.

We viewed the main advantage of age-specific tests of association as being that the two measurements are closer in time. This would make the tests more sensitive if elevated NAA itself has a direct, time-sensitive influence on cognitive performance. We viewed the main advantage of correlations using NAA data across all ages as being that the larger set of NAA measurements available for each animal yields a less noisy estimate of the underlying MIA-induced effect. This would make the tests of association more sensitive if elevated NAA is indirectly associated with cognitive performance through an underlying and ongoing adaptive process induced by MIA. The latter scenario is more consistent with our general model, which is that elevated NAA is more likely to be indirectly rather than directly related to cognitive performance in these animals. That is, we view ongoing elevation of NAA in the MIA animals as one manifestation of an underlying adaptive process that also leads to preserved cognitive function. We do not view elevated NAA itself is the cause of preserved cognition. This was our rationale for using NAA data across all ages rather than age-specific NAA data for the correlations with cognitive measures.

Table S1. Estimated differences and standard errors from the linear mixed-effects models, assessing the relationship between group and prefrontal brain metabolites measures, not adjusted by gray matter tissue fraction.

	Estimated difference between MIA and Control[1]											
	Overall group difference		Age-specific group difference									
			6 Months		12 Months		24 Months		36 Months		45 Months	
**Outcome**	***Estimate (SE)***	***P-value***	***Estimate (SE)***	**P-value**	***Estimate (SE)***	**P-value**	***Estimate (SE)***	**P-value**	***Estimate (SE)***	**P-value**	***Estimate (SE)***	**P-value**
**Normalized by partial volume corrected water**												
NAA	**0.37 (0.10)**	**<.001**	-	-	-	-	-	-	-	-	-	-
Inositol	**-0.31 (0.14)**	**.037**	-	-	-	-	-	-	-	-	-	-
Glutamate	0.02 (0.17)	.92	-	-	-	-	-	-	-	-	-	-
Choline	0.01 (0.03)	.67	-	-	-	-	-	-	-	-	-	-
Creatine	-	-	-.07 (0.15)	.62	0.03 (0.15)	.83	0.08 (0.14)	.57	-0.27 (0.14)	.067	-0.27 (0.15)	.070
Taurine	-	-	-0.14 (0.09)	.14	0.02 (0.09)	.81	0.17 (0.09)	.063	**0.32 (0.09)**	**.001**	**0.20 (0.09)**	**.034**
GSH	-49.2 (119.2)	.68	-	-	-	-	-	-	-	-	-	-
**Normalized by creatine**												
NAA	**0.07 (0.02)**	**.005**	-	-	-	-	-	-	-	-	-	-
Inositol	-0.03 (0.02)	.086	-	-	-	-	-	-	-	-	-	-
Glutamate	-	-	0.07 (0.04)	.065	-0.01 (0.04)	.73	0.00 (0.04)	.94	0.05 (0.04)	.14	0.07 (0.04)	.062
Choline	0.004 (0.004)	.37	-	-	-	-	-	-	-	-	-	-
Taurine	-	-	-0.01 (0.01)	.29	0.00 (0.01)	.77	0.02 (0.01)	.099	**0.05 (0.01)**	**<.001**	**0.04 (0.01)**	**.006**
GSH	0.001 (0.002)	.62	-	-	-	-	-	-	-	-	-	-

Abbreviations: MIA = maternal immune activation, SE = standard error, NAA= n-acetylaspartate, GSH = glutathione.

[1]Estimated differences and *P*-values for prefrontal brain metabolites measures from linear mixed-effects models that included fixed effects for group (MIA, Control), age at testing (6, 12, 24, 36, 45 months), and their interaction, with covariance structure to account for within-animal correlation (spatial exponential correlation for NAA normalized by partial volume corrected water and creatine-normalized GSH, unstructured

covariance for glutamate normalized by partial volume corrected water, and compound symmetry correlation for other outcomes). The interaction was removed from the final model if it was not significant. If interaction of group with age was significant, estimated age-specific group differences were reported. If the interaction of group with age was not significant, the estimated group difference was the same across all ages (hence only overall group difference was reported for all ages).

Table S2. Summary for creatine-normalized prefrontal brain metabolite measures.

	6 Months		12 Months		24 Months		36 Months		45 Months	
	MIA[1] (*n* = 14)	Control (*n* = 13)	MIA[2] (*n* = 13)	Control (*n* = 14)	MIA[3] (*n* = 13)	Control[4] (*n* = 14)	MIA[3] (*n* = 13)	Control[3] (*n* = 14)	MIA[3] (*n* = 12)	Control[3] (*n* = 14)
Age (days), *mean* (*SD*) [*Range*]										
	179.9 (1.6) [177-182]	181.2 (5.5) [177- 199]	365.5 (1.5) [364-369]	365.1 (0.7) [364-366]	730.1 (1.3) [729-733]	729.8 (1.0) [729-732]	1094.6 (1.0) [1093-1096]	1098.9 (8.2) [1092-1116]	1371.6 (1.9) [1368-1374]	1370.9 (1.6) [1368-1374]
Brain metabolite values (normalized by creatine), *mean* (*SD*)										
NAA	1.12 (0.06)	1.05 (0.07)	1.04 (0.09)	0.99 (0.07)	1.07 (0.12)	1.01 (0.07)	1.09 (0.09)	1.01 (0.07)	1.14 (0.10)	1.07 (0.06)
Inositol	0.88 (0.07)	0.90 (0.04)	0.82 (0.07)	0.87 (0.06)	0.82 (0.07)	0.86 (0.04)	0.80 (0.07)	0.83 (0.04)	0.79 (0.05)	0.80 (0.05)
Glutamate	1.65 (0.09)	1.58 (0.11)	1.46 (0.10)	1.48 (0.11)	1.37 (0.10)	1.38 (0.07)	1.37 (0.11)	1.32 (0.08)	1.41 (0.14)	1.34 (0.06)
Choline	0.20 (0.02)	0.20 (0.02)	0.19 (0.02)	0.18 (0.01)	0.19 (0.01)	0.19 (0.01)	0.19 (0.01)	0.18 (0.01)	0.19 (0.02)	0.18 (0.02)
Taurine	0.28 (0.03)	0.30 (0.03)	0.19 (0.04)	0.19 (0.03)	0.16 (0.03)	0.14 (0.02)	0.18 (0.05)	0.13 (0.02)	0.16 (0.04)	0.12 (0.02)
GSH	0.05 (0.02)	0.05 (0.01)	0.05 (0.01)	0.05 (0.01)	0.04 (0.01)	0.04 (0.01)	0.05 (0.01)	0.05 (0.01)	0.05 (0.02)	0.05 (0.01)

Note: MIA = maternal immune activation, SD = standard deviation, NAA= n-acetylaspartate, GSH = glutathione.

[1]Some values were missing: 2 for NAA, 2 for Inositol, 2 for Glutamate, 3 for Taurine, 3 for Choline.

[2]One value was missing for all metabolites except GSH.

[3]Taurine value was missing for 1 animal.

[4]Taurine value was missing for 2 animals.

Table S3. Group differences in creatine-normalized prefrontal brain metabolites from the linear mixed-effects models, adjusted by gray matter tissue fraction.

	Estimated difference between MIA and Control[1]											
	**Overall group difference**		**Age-specific group difference**									
			**6 Months**		**12 Months**		**24 Months**		**36 Months**		**45 Months**	
**Outcome**	***Estimate (SE)***	***P-value***	***Estimate (SE)***	***P-*****value**	***Estimate (SE)***	***P-*****value**	***Estimate (SE)***	***P-*****value**	***Estimate (SE)***	***P-*****value**	***Estimate (SE)***	***P-*****value**
NAA	**0.07 (0.02)**	**.005**	-	-	-	-	-	-	-	-	-	-
Inositol	-0.03 (0.02)	.085	-	-	-	-	-	-	-	-	-	-
Glutamate	-	-	**0.07 (0.04)**	**.043**	-0.01 (0.03)	.76	-0.01 (0.03)	.84	0.05 (0.03)	.16	**0.08 (0.04)**	**.033**
Choline	0.003 (0.005)	.50	-	-	-	-	-	-	-	-	-	-
Taurine	-	-	-0.01 (0.01)	.45	0.01 (0.01)	.65	0.01 (0.01)	.27	**0.04 (0.01)**	**<.001**	**0.04 (0.01)**	**<.001**
GSH	0.001 (0.003)	.67	-	-	-	-	-	-	-	-	-	-

Abbreviations: MIA = maternal immune activation, SE = standard error, NAA= n-acetylaspartate, GSH = glutathione.

[1]Estimated differences and *P*-values for brain metabolites measures from linear mixed-effects models that included fixed effects for group (MIA, Control), age at testing (6, 12, 24, 26, 45 months), their interaction, and tissue fraction gray matter, with covariance structure to account for within-animal correlation (compound symmetry correlation for all outcomes). The interaction was removed from the final model if not significant. If interaction of group with age was significant, estimated age-specific group differences were reported. If the interaction of group with age was not significant, the estimated group difference was the same across all ages (hence only overall group difference was reported for all ages).

Figure S1

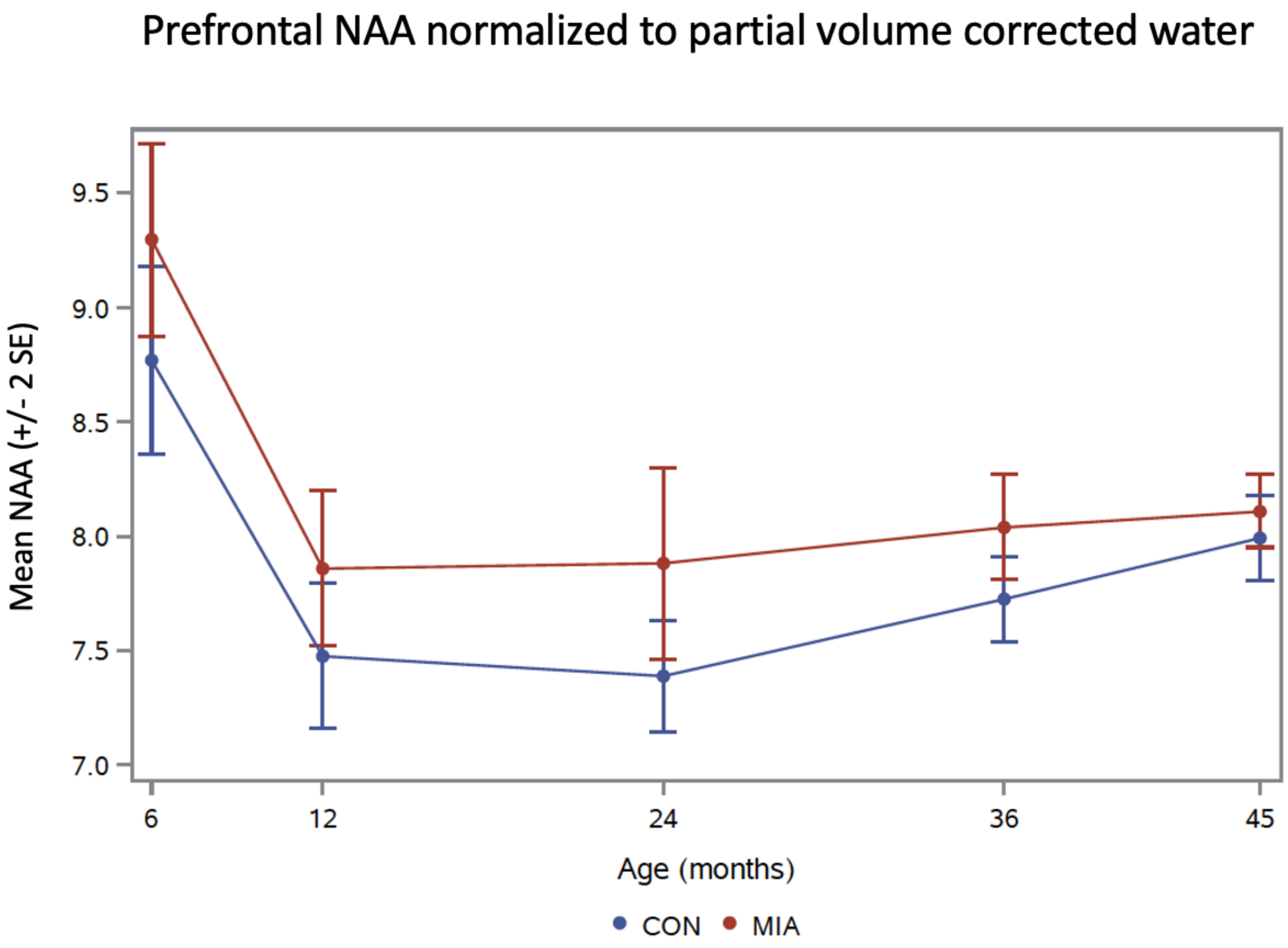

Con = Control offspring; MIA = Maternal Immune Activation offspring

Figure S2

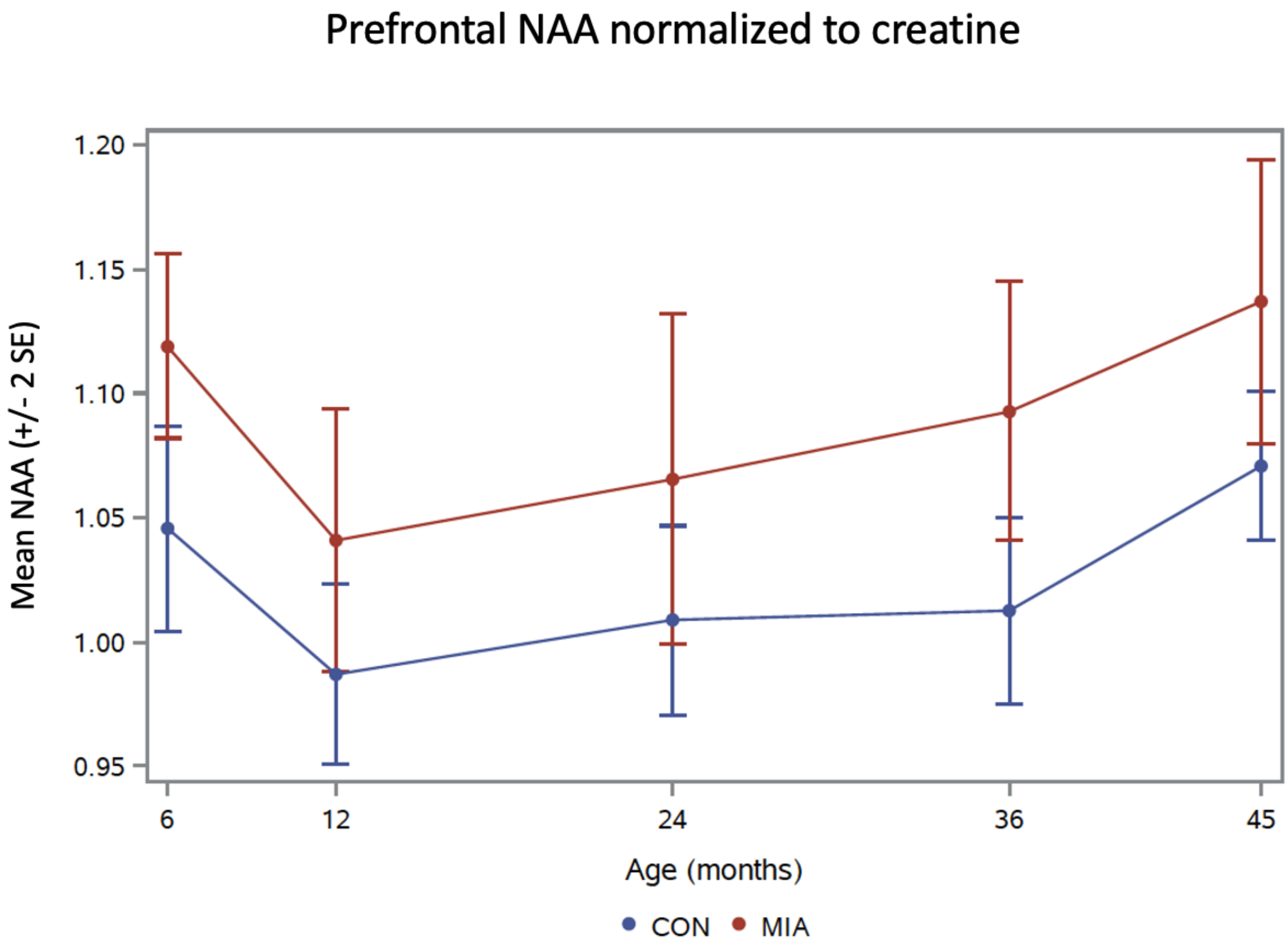

Con = Control offspring; MIA = Maternal Immune Activation offspring

Figure S3 - Individual NAA values at each age for each animal

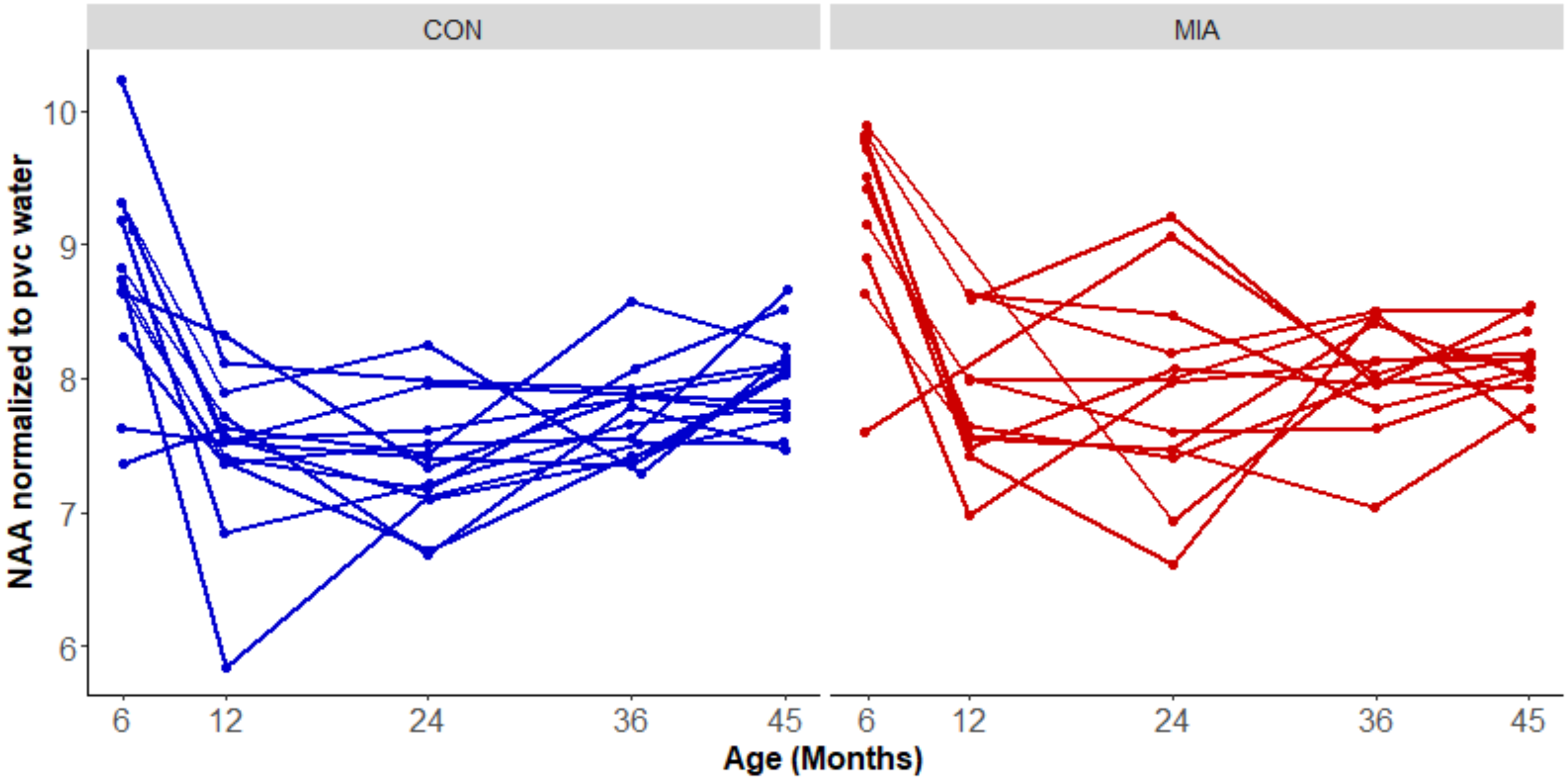

MIA = Maternal Immune Activation offspring; Con = Control offspring; pvc = partial volume corrected.

Figure S4

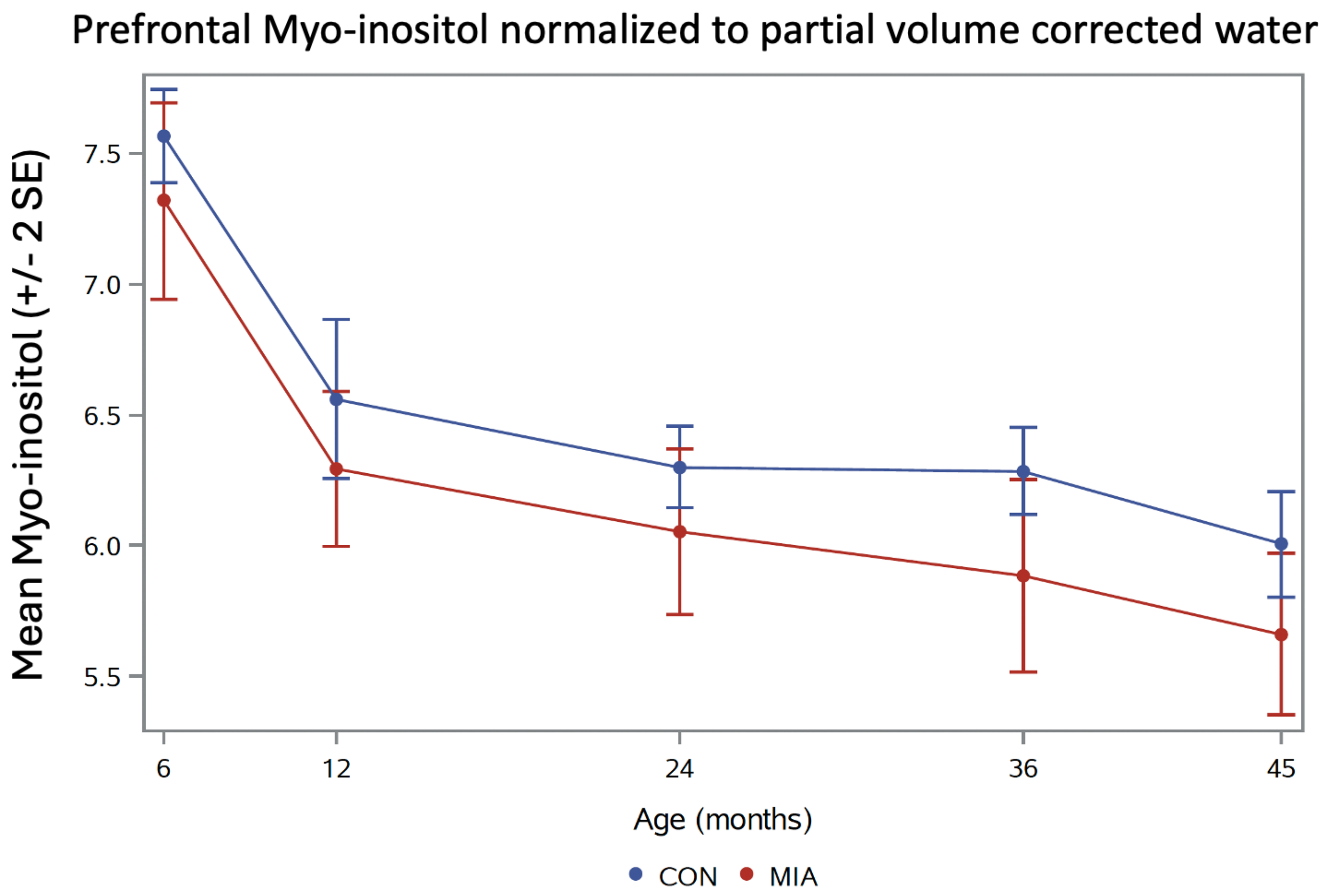

Con = Control offspring; MIA = Maternal Immune Activation offspring

Figure S5 - Individual Myo-inositol values at each age for each animal

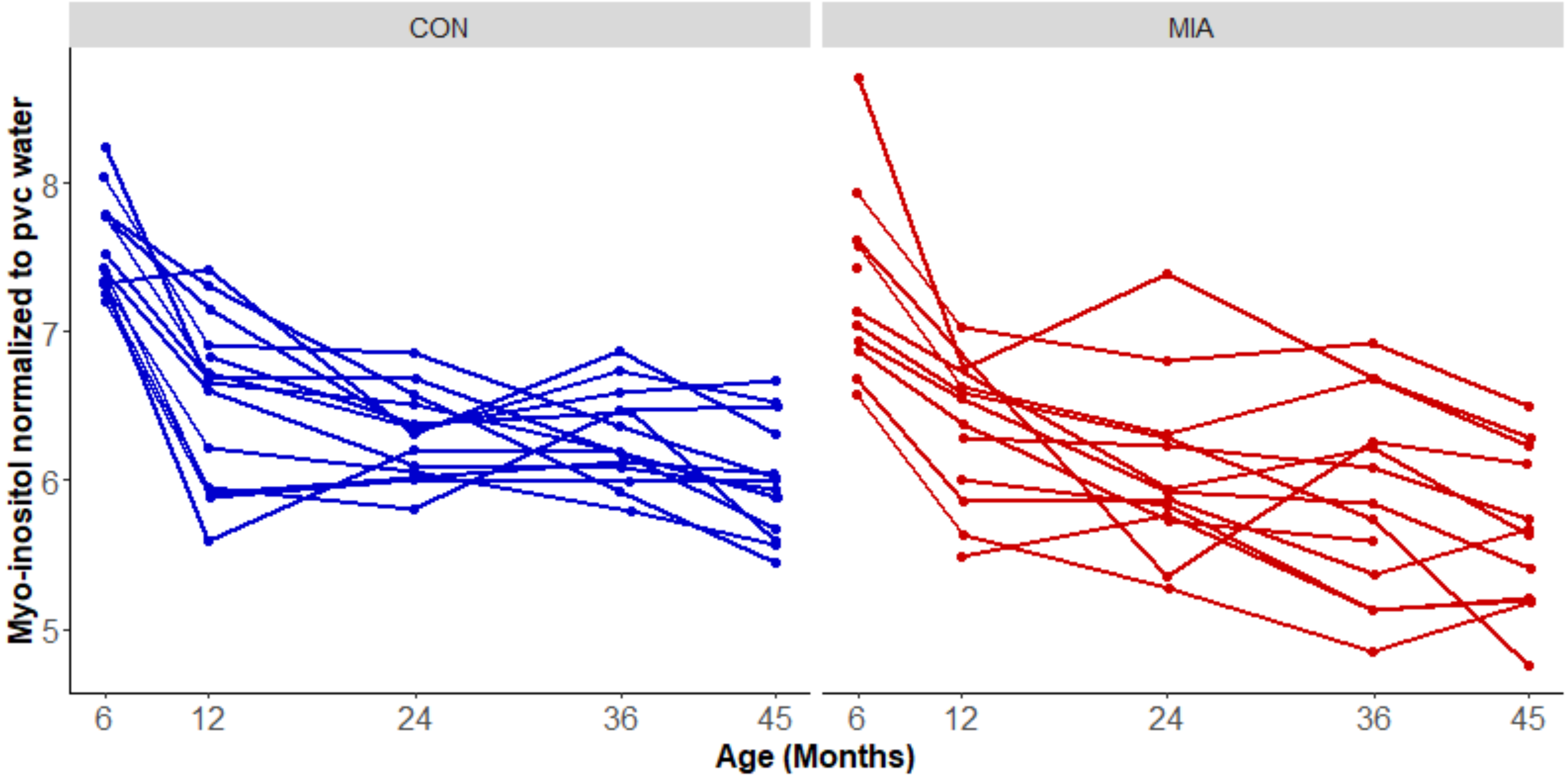

MIA = Maternal Immune Activation offspring; Con = Control offspring; pvc = partial volume corrected.

Figure S6

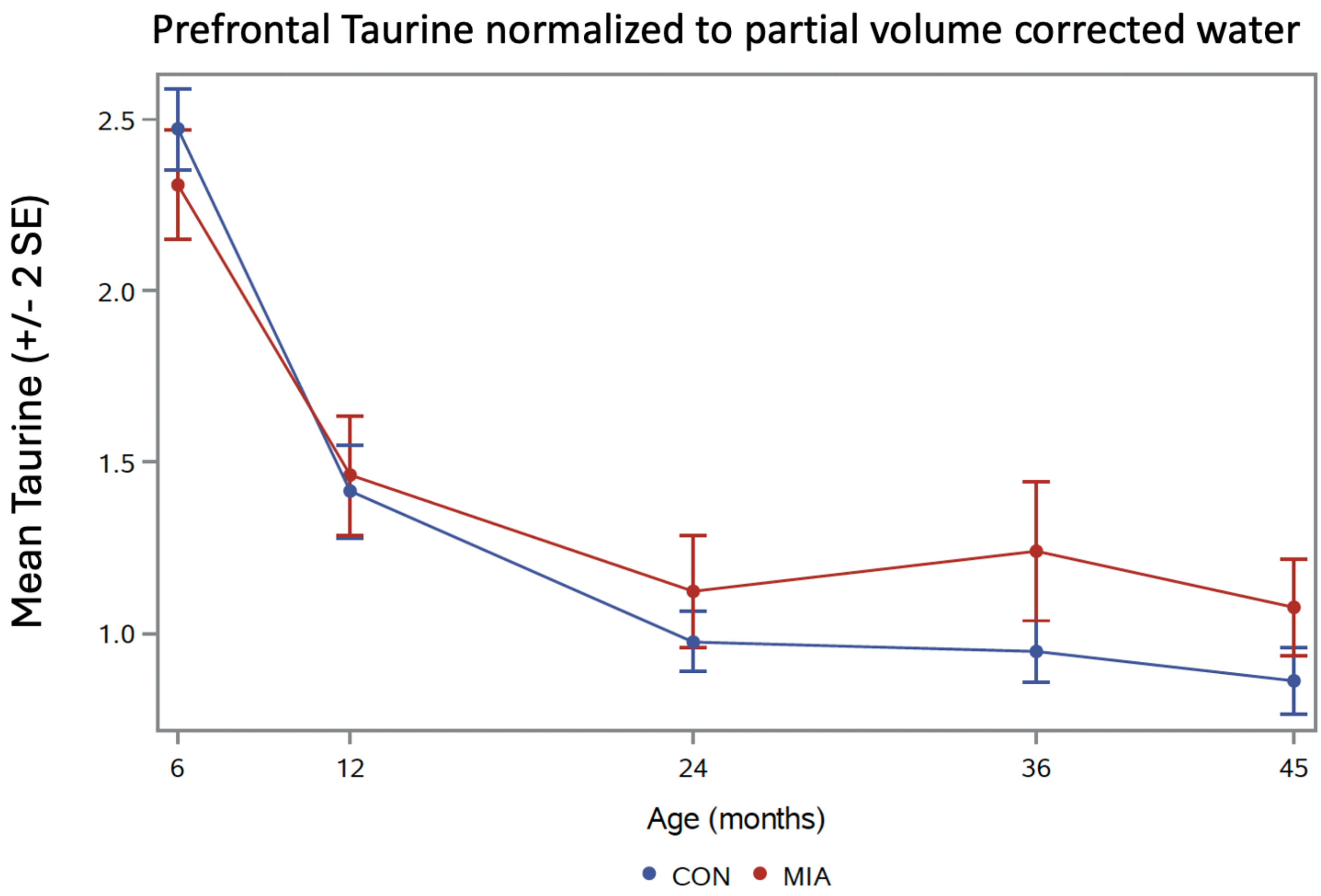

Con = Control offspring; MIA = Maternal Immune Activation offspring

Figure S7 - Individual Taurine values at each age for each animal

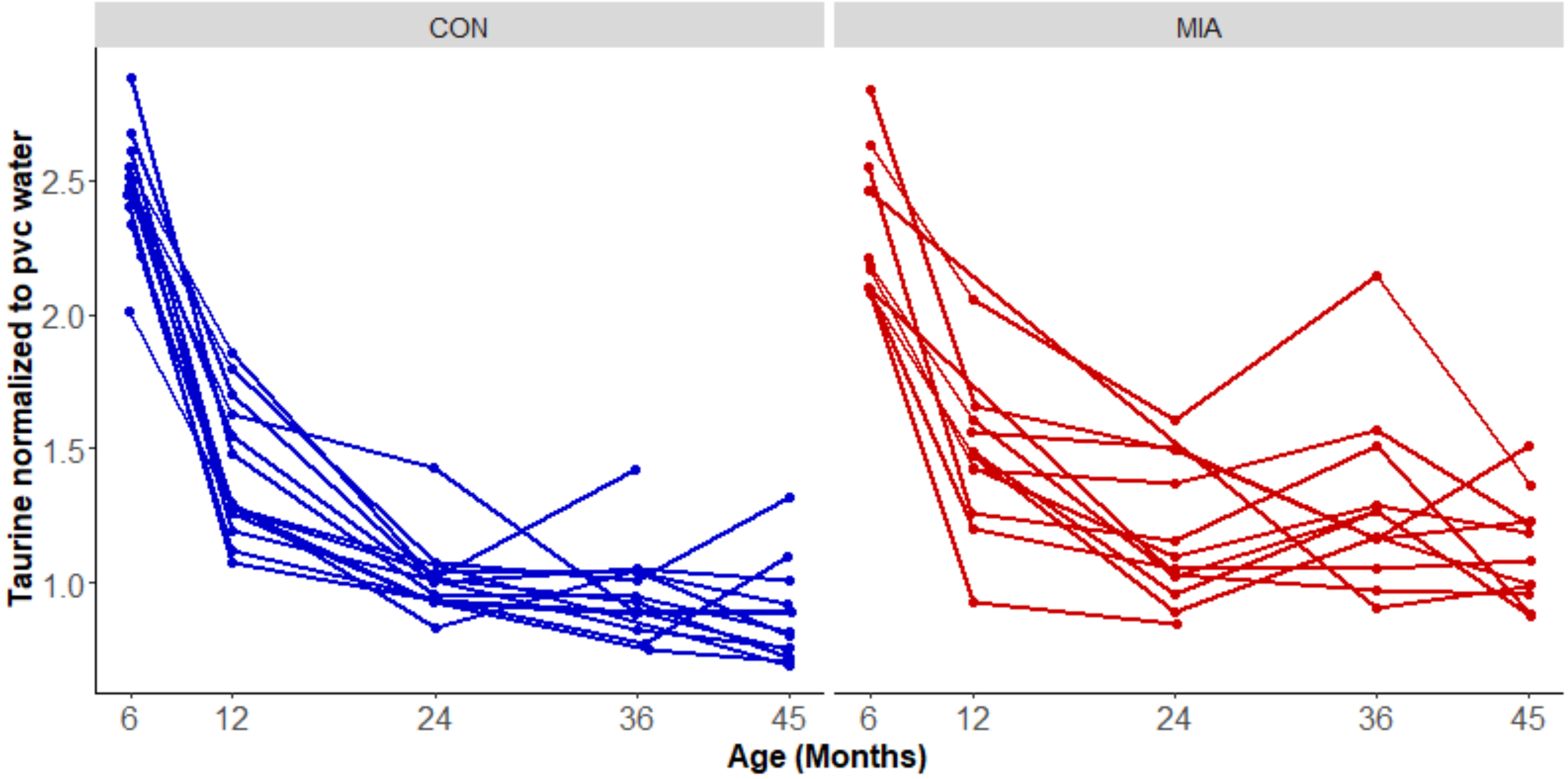

MIA = Maternal Immune Activation offspring; Con = Control offspring; pvc = partial volume corrected.

Figure S8

NAA is inversely associated with Reversal Learning errors at age 21 Months in MIA group

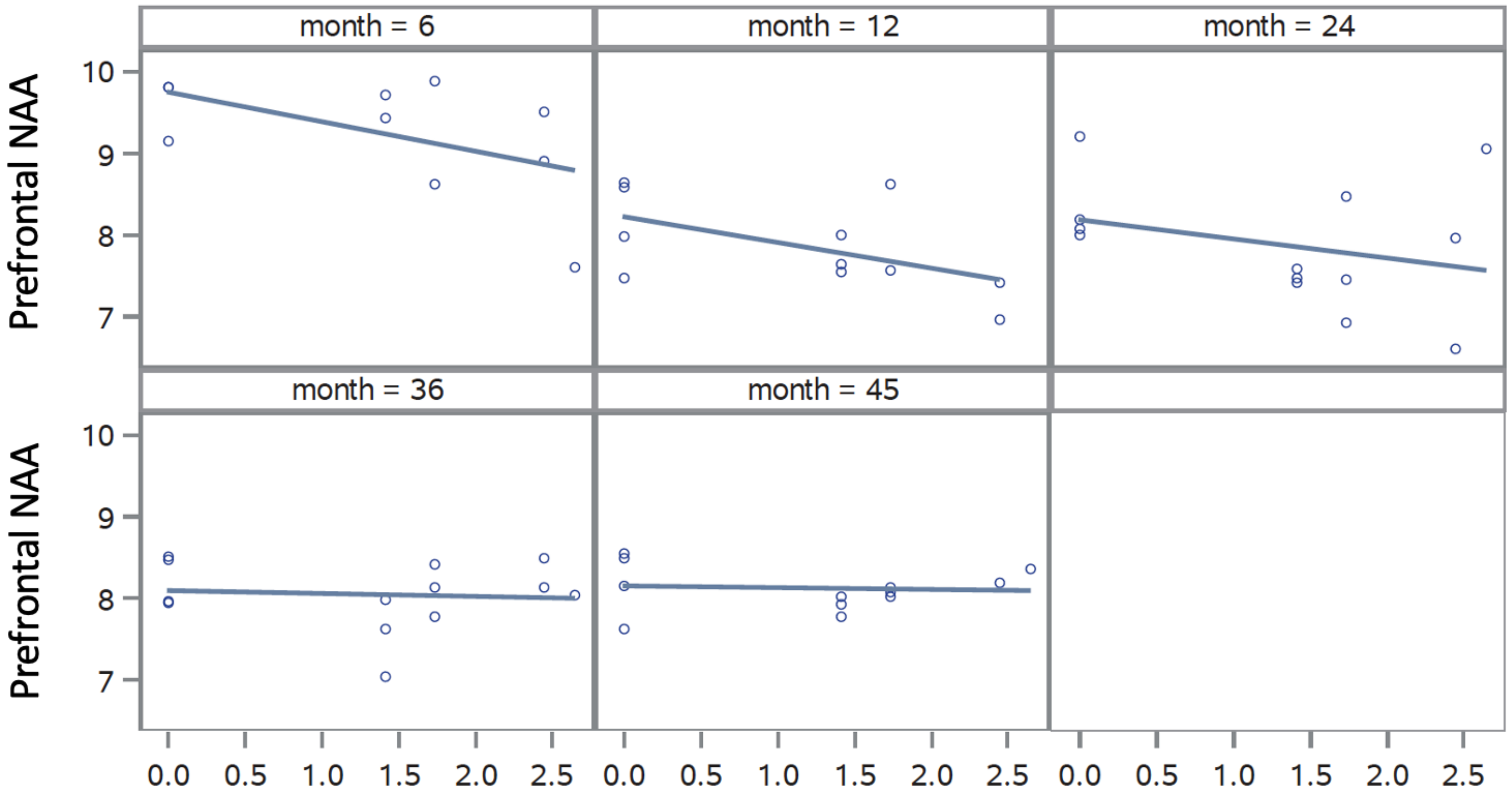

NAA is normalized by partial volume corrected water. X-axis is square root of omission errors on Reversal Learning at age 21 months. NAA data shown is from MIA group only at each age. Solid line is regression line. A Linear Mixed-effects Model analysis shows a significant inverse relationship between NAA (across all ages) and Reversal Learning omission errors at 21 months (p = .023, see Table 3). Note that association is strongest for ages up to the time of testing at 21 months. MIA = Maternal Immune Activation offspring.

Figure S9

NAA is inversely associated with miss rate on Simple Discrimination Reversal (SDR) touchscreen task at age 46 – 47 Months in MIA group

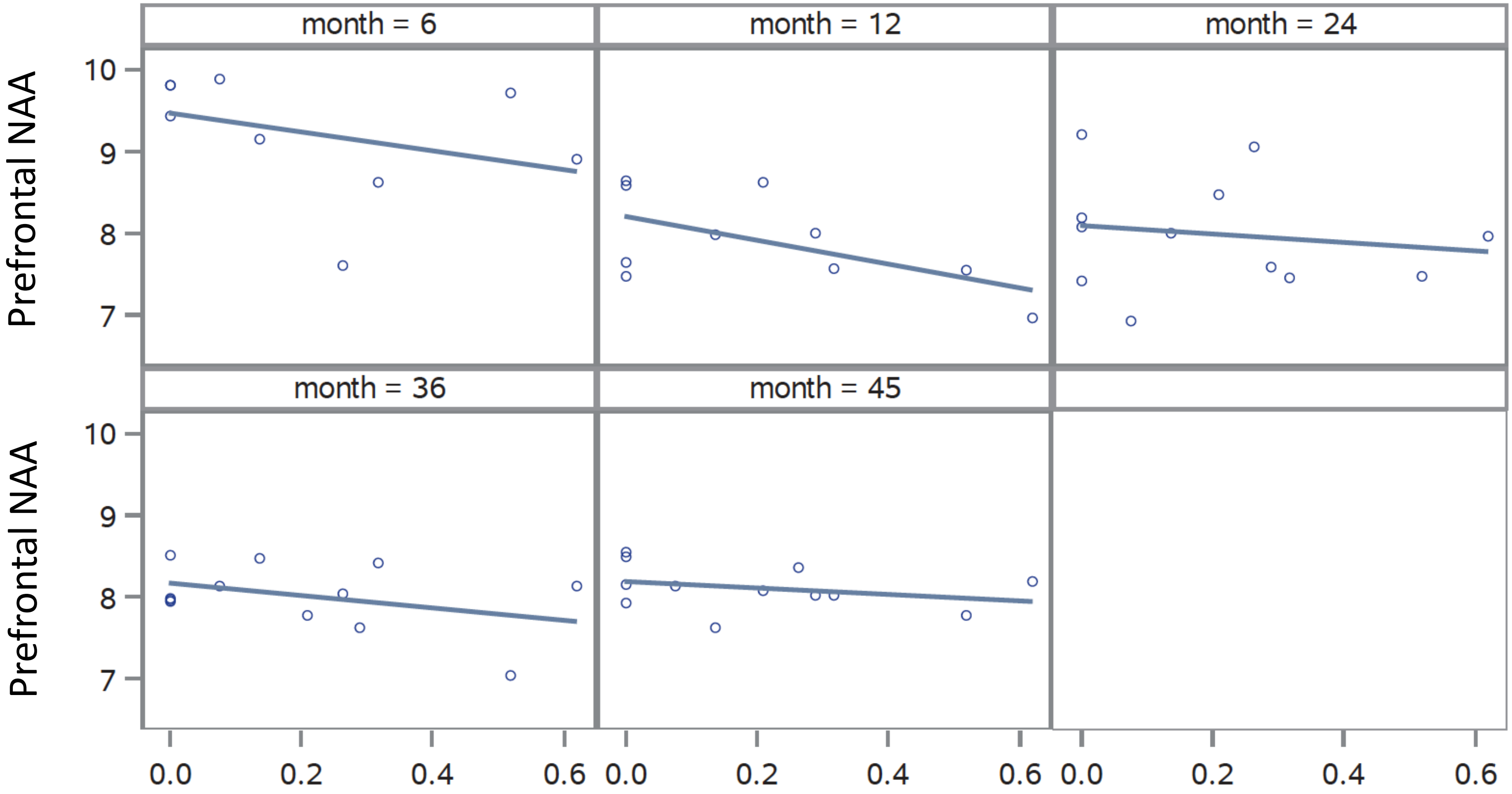

NAA is normalized by partial volume corrected water. X-axis is square root of miss rate on SDR touchscreen task at age 46 - 47 months. NAA data shown is from MIA group only at each age. Solid line is regression line. A Linear Mixed-effects Model analysis shows a significant inverse relationship between NAA (across all ages) and SDR miss rate at age 46– 47 months ($p = .031$, see Table 3). MIA = Maternal Immune Activation offspring.
